# Immunometabolic actions of trabectedin and lurbinectedin on human macrophages: relevance for their anti-tumor activity

**DOI:** 10.3389/fimmu.2023.1211068

**Published:** 2023-08-22

**Authors:** Adrián Povo-Retana, Marco Fariñas, Rodrigo Landauro-Vera, Marina Mojena, Carlota Alvarez-Lucena, Miguel A. Fernández-Moreno, Antonio Castrillo, Juan Vladimir de la Rosa Medina, Sergio Sánchez-García, Carles Foguet, Francesc Mas, Silvia Marin, Marta Cascante, Lisardo Boscá

**Affiliations:** ^1^ Instituto de Investigaciones Biomédicas Alberto Sols, CSIC-UAM, Madrid, Spain; ^2^ Department of Biochemistry and Molecular Biomedicine-Institute of Biomedicine (IBUB), Faculty of Biology, Universitat de Barcelona, Barcelona, Spain; ^3^ Departamento de Bioquímica, Facultad de Medicina, Universidad Autónoma de Madrid, Madrid, Spain; ^4^ Unidad de Biomedicina (Unidad Asociada al CSIC) de la Universidad de Las Palmas de Gran Canaria, Las Palmas, Spain; ^5^ Unidad Instituto Universitario de Investigaciones Biomédicas y Sanitarias (IUIBS) de la Universidad de Las Palmas de Gran Canaria, Las Palmas, Spain; ^6^ British Heart Foundation Cardiovascular Epidemiology Unit, Department of Public Health and Primary Care, University of Cambridge, Cambridge, United Kingdom; ^7^ Department of Material Science and Physical Chemistry & Research Institute of Theoretical and Computational Chemistry (IQTCUB), University of Barcelona, Barcelona, Spain; ^8^ CIBER of Hepatic and Digestive Diseases (CIBEREHD), Institute of Health Carlos III (ISCIII), Madrid, Spain; ^9^ Centro de Investigación Biomédica en Red de Enfermedades Cardiovasculares (CIBERCV), Av. Monforte de Lemos, Madrid, Spain

**Keywords:** macrophages, immunometabolism, trabectedin, lurbinectedin, ROS

## Abstract

In recent years, the central role of cell bioenergetics in regulating immune cell function and fate has been recognized, giving rise to the interest in immunometabolism, an area of research focused on the interaction between metabolic regulation and immune function. Thus, early metabolic changes associated with the polarization of macrophages into pro-inflammatory or pro-resolving cells under different stimuli have been characterized. Tumor-associated macrophages are among the most abundant cells in the tumor microenvironment; however, it exists an unmet need to study the effect of chemotherapeutics on macrophage immunometabolism. Here, we use a systems biology approach that integrates transcriptomics and metabolomics to unveil the immunometabolic effects of trabectedin (TRB) and lurbinectedin (LUR), two DNA-binding agents with proven antitumor activity. Our results show that TRB and LUR activate human macrophages toward a pro-inflammatory phenotype by inducing a specific metabolic rewiring program that includes ROS production, changes in the mitochondrial inner membrane potential, increased pentose phosphate pathway, lactate release, tricarboxylic acids (TCA) cycle, serine and methylglyoxal pathways in human macrophages. Glutamine, aspartate, histidine, and proline intracellular levels are also decreased, whereas oxygen consumption is reduced. The observed immunometabolic changes explain additional antitumor activities of these compounds and open new avenues to design therapeutic interventions that specifically target the immunometabolic landscape in the treatment of cancer.

## Highlights

- TRB and LUR trigger pro-inflammatory pathways in resistant hMφ
- Mitochondrial and cytoplasmic ROS are produced in response to TRB and LUR
- TRB and LUR promote mitochondrial biogenesis, repress OXPHOS and interfere TCA
- RNAseq analysis shows that TRB and LUR upregulate MHC class I expression
- TRB and LUR transcriptionally activate glycolysis and PPP pathways

## 1 Introduction

Recent advances in immunometabolism have unveiled the key role of different metabolites as signaling molecules either for immune cells, to support the activation, or for pathogens to reshape their ligand repertoire to scape/subvert immune cells (1).

Macrophages are innate immune cells that mediate the removal of pathogens or damaged cells (2). In particular, immunometabolic signatures associated with macrophage polarization have been extensively characterized in the last few years (3). The amplification of the innate response is regulated by the secretion of pro-inflammatory mediators and chemokines that target several cell types, including monocytes, other macrophages, natural killer cells, neutrophils, and epithelial and endothelial cells. Typical pro-inflammatory mediators include interleukin- (IL-) 1β, IL-6, IL-12, tumor necrosis factor-α (TNF-α), chemokine (C-C motif) ligand (CCL) 2, and CCL4.

Acquisition of either an inflammatory or anti-inflammatory phenotype by macrophages ultimately depends on the microenvironment at the site of inflammation. Therefore, when a pathogen or a stressed cell is to be cleared, macrophages are activated toward an inflammatory profile (M1 phenotype) (3), followed by a gradual switch into an anti-inflammatory/pro-resolving profile (M2 phenotype) (4). At the metabolic level, M1 macrophages rely on aerobic glycolysis and the pentose phosphate pathway (PPP) to meet their energetic demands, whereas M2 macrophages are more dependent on oxidative phosphorylation, using glucose as an electron supplier through canonical glycolysis and the tricarboxylic acid (TCA) cycle (5). M1 macrophages also exhibit an altered TCA cycle, which is disrupted at several steps, leading to citrate and succinate export into the cytoplasm, where these metabolites play several regulatory roles that do not occur in M2-macrophages. Cytoplasmic succinate activates the hypoxia-inducible factor 1-α (HIF1α), a key M1-polarizing transcription factor (6). Additionally, α-ketoglutarate (α-KG) is a repressor of M1 polarization (and increases M2 polarization) by inhibiting the activity of the NF-κB transcription factor (7). Thus, the succinate/α-KG ratio provides information on the M1 *vs*. M2 polarization. It has been postulated that increased glycolytic flux in M1 macrophages favors the deviation of the triose-phosphate glycolytic intermediates toward methylglyoxal (MG) production (8). This increase in MG can exceed the capacity of the glyoxalase (GLO) pathway that clears MG through its conversion into D-lactate *via* two sequential reactions in which MG is transformed into *S*-D-lactoylglutathione (GLO1), and finally into D-lactate by GLO2.

In this context, we have investigated the immunometabolic signatures of the antitumoral drugs trabectedin (TRB) (9, 10) and lurbinectedin (LUR) (9). TRB is indicated for the treatment of advanced soft-tissue sarcoma in adults, and in combination with pegylated liposomal doxorubicin for patients with relapsed platinum-sensitive ovarian cancer (9, 11). LUR, a structural analog of TRB, is indicated for the treatment of metastatic small-cell lung cancer (12, 13). Both drugs act as DNA-binding agents that inhibit activated transcription, affecting the ability of oncogenic transcription factors to bind within their recognition sequences. It is known that the recognition of LUR is concentrated in GC-rich areas within the promoters (14). Additionally, LUR inhibits active transcription through the specific and rapid degradation of elongating RNA polymerase II by the ubiquitin-proteasome machinery (15). Both drugs induce delayed transition through phase S of the cell cycle and a final arrest in the phases G2/M, triggering tumor cell death by apoptosis.

TRB and LUR are known to modulate the immune response within the tumor microenvironment by specifically targeting mononuclear phagocytes (16–18). Previously, we characterized the biological response of these drugs in human macrophages (hMφ) (15). Here, we report that treatment of hMφ from healthy donors with TRB or LUR results in two different behaviors in terms of cell viability: those that exhibited a rapid induction of apoptotic death, and those that retained viability, even at supratherapeutic doses of the drugs (15). Here, we present evidence that these drugs trigger the resistant hMφ toward a pro-inflammatory functional phenotype by inducing a common specific metabolic rewiring program and using Genome-Scale Metabolic Modeling (GSMM) methods (19–21) we identified targetable pathways and mechanisms that show promising potential as adjuncts for combined therapies. These observations highlight the profound impact of coordinated metabolic networks on the outcome of the macrophage-drug interaction and open novel avenues for the rational design of new therapies.

## 2 Materials and methods

### 2.1 Materials

Common reagents were from Sigma-Aldrich-Merck (Madrid, Spain) or Roche (Darmstadt, Germany). Human cytokines were from PeproTech (London, UK) or Merck. Tissue culture dishes were from Falcon (Lincoln Park, NJ, USA), and serum and culture media were from Invitrogen (Life Technologies/Thermo-Fisher, Madrid, Spain).

### 2.2 Administration of the antitumor drugs trabectedin and lurbinectedin

Drugs were used at low nanomolar contentrations (0-100 nM). Final assay dilutions were prepared from a 10 µM stock in DMSO, and diluted in RPMI 1640 and 2% FBS media (Sigma). Solid compounds were periodically provided by PharmaMar (Colmenar Viejo, Spain) (22).

### 2.3 Isolation of human monocytes and preparation of human macrophages

Cells were prepared from buffy coats obtained from anonymous healthy donors in agreement with the Institutional and Centro de Transfusiones de la Comunidad de Madrid agreements (28504/000011). Donors were informed and provided written consent following the ethical guidelines of the 1975 Declaration of Helsinki and the Committee for Human Subjects.

To isolate human peripheral blood mononuclear cells (PBMC), buffy coats were treated with Ficoll (17-0300, Sigma-Aldrich-GE) by carefully adding blood by soft dripping to prevent the two-phase mixture and then centrifuged for 25 min at 450 ***g*** at RT without brake. Plasma and PBMC fractions were collected from the upper-aqueous phase of the Ficoll gradient and washed twice with sterile PBS by centrifuging for 5 min at 300 ***g*** at RT. Remnant erythrocytes from PBMC fraction were lysed by adding diluted red blood cell lysis buffer 10x (420302, Biolegend) followed by washing with sterile PBS twice. Cell count and viability were evaluated by flow cytometry (Cytoflex-S, Becton Dickinson) and trypan blue (T8154, Sigma). Finally, PBMC were centrifuged for 8 min at 300 ***g*** at RT, resuspended in FBS-free DMEM (41966-029, Gibco) with penicillin/streptomycin (15140/122, Gibco) and seeded at 2x10^6^ cells/well in 6-well cell culture plates (353046, Falcon).

Human macrophages (hMφ) were prepared after culture in FBS-free DMEM for 1h to induce monocyte cell adhesion. Then, 10% heat-inactivated FBS (10270/106, Gibco) was added to the cell media and left overnight. Cells were washed twice with sterile PBS to remove lymphocytes and culture media was replaced with DMEM and 10% heat-inactivated FBS. Cells were incubated for 7 days, allowing human monocytes differentiation into hMφ. After differentiation, the culture medium was replaced with RPMI1640 (21875, Gibco) and FBS 2% 18h before the experiments. CD14^+^-cells were >90%

### 2.4 hMφ polarization assays

hM1 polarization (23) was performed by incubating for 24h hMφ with the following bioactive molecules: LPS (0.5 µg/mL; LPS-EB Ultrapure, InvivoGen, 5×10^6^ EU, tlrl-3pelps, Ibian Technologies, Zaragoza, Spain), human recombinant IL-1β (PeproTech, 200-01B; 20 ng/mL, London, UK) and recombinant human IFN-γ (PeproTech, 300-02; 20 ng/mL), and TNF-α (PeproTech, 300-01A; 20 ng/mL), followed by challenging with either TRB or LUR. hM2 polarization was carried out by incubating for 24h hMφ with the following combination of human recombinant cytokines: IL-4 (PeproTech, 200-04; 20 ng/mL), IL-10 (PeproTech, 200-10; 20 ng/mL), and IL-13 (PeproTech, 200-13; 20 ng/mL).

### 2.5 Flow cytometry assays

Flow cytometry experiments were carried out in a Cytoflex S (Becton Dickinson, Madrid, Spain). Differentiated hMφ supernatants were preserved, cells were trypsinized for 4 min at 37 °C and 5% CO_2_, and trypsin was neutralized with sterile PBS + 2% FBS. Cells were gently scraped and centrifuged at 300 ***g***, at room temperature for 5 min. Cells were then incubated with different fluorochromes for 30 min (unless indicated otherwise). Cell media supernatants were always centrifuged and properly considered for all cell viability determinations. Cell viability was determined by DAPI staining (2 µM, Life Technologies, Madrid, Spain) and incubating for 5 min at room temperature (15). Experiments were analyzed using CytExpert software.

### 2.6 Seahorse measurements (Agilent Technologies XF24)

Oxygen consumption rate (OCR) was measured in real-time, following the instructions of the manufacturer (Agilent, 103576-100, Madrid, Spain). The seahorse analyzer was calibrated with a calibrating Seahorse XF solution (Agilent, 103059-000). Respiratory chain inhibitors were used at these concentrations: 6 μM oligomycin, 0.75 mM DNP (2′,4′-dinitrophenol), 1 μM rotenone, and 1 μM antimycin A (15, 23).

### 2.7 Measurement of ROS production

ROS production was measured by incubating cells for 30 min at 37 °C and 5% CO_2_ in darkness with 5 µM DCFH-DA fluorescent probe (2′-7′-dichlorofluorescein diacetate; D6683, Sigma). The oxidation of DCFH was quantified by flow cytometry (24–26). For O_2_ mitochondrial superoxide species measurements, 5 µM MitoSOX (Invitrogen, Ref M36008) were incubated with hMφ for 30 min at 37 °C and 5% CO_2_ in darkness (22).

### 2.8 Evaluation of mitochondrial inner membrane potential

Mitochondrial membrane potential (ΔΨm) measures in hMφ were monitored by 100 nM CMXRos (Red MitoTracker; M7512, Invitrogen). The fluorescent probe was incubated for 30 min at 37°C and 5% CO_2_ in darkness, following previous protocols (27).

### 2.9 Mitochondrial mass determination

Mitochondrial mass measures in hMφ-resistant (hMφ-R) were monitored by 100 nM mitogreen (MitoTracker Green; M7514, Invitrogen). The fluorescent probe was incubated for 30 min at 37 °C and 5% CO_2_ in darkness.

### 2.10 Neutral lipid content measurements

Neutral lipid content was assessed by 1 µM Bodipy (Invitrogen.....). The cells were incubated with the fluorescent probe for 10 min at 37 °C and 5% CO_2_ in darkness and analyzed by flow cytometry.

### 2.11 hMφ-R lysosomal trafficking assays

A Lysotracker™ Red DND-99 fluorescent probe was used (Life Technologies, L7528, 1µM). In this case, cells were trypsinized and scrapped off the plate as described above and exposed to this specific fluorescent FITC-labelled compound for 30 min at 37°C + 5% CO_2_ in darkness. Cells were washed twice with sterile PBS and then measured in Cytoflex S.

### 2.12 RNA Isolation and analysis

RNA from cells was extracted in Trizol Ambion (AM9738, Thermo Fisher) following the manufacturer’s instructions. RNA was quantified in a NanoDrop 2000 (ThermoFisher) and 1 µg RNA was reverse-transcribed to cDNA with Transcriptor First-Strand cDNA Synthesis kit (04379012001, Roche). qPCR assay was carried out with 5 µL of this template cDNA, 10 µL SYBR Green PCR Master Mix cocktail (4309155, ThermoFisher) and 250 nM forward and reverse primers (**Supplementary Table S2**). *RPLP0* (*36B4*) was chosen as a housekeeping endogenous control for normalization purposes. qPCR reaction was carried out in MyIQ RealTime PCR System (BioRad). Result analysis was conducted with the IQ5 program (BioRad) following the 2^-ΔΔCt^ method.

### 2.13 RNA integrity determination

Agilent 2100 Bioanalyzer device provided a framework for the standardization of RNA quality control. RNA samples were separated by electrophoresis on a micro-fabricated chip and subsequently detected via laser-induced fluorescence detection. The use of an RNA ladder as a mass and size standard during electrophoresis allowed the estimation of the RNA band sizes and relative quantities. RNA quality assessment is the RNA Integrity Number (RIN) that depends on the shape of the curve obtained in the electropherogram. The software and the algorithm allow the classification of total RNA on a numbering system from 1 to 10, with 1 being the most degraded profile and 10 being the most intact.  RNA samples were considered preserved, 8≤RIN samples≤10. If RIN was not 8 or higher, samples were not considered qualified and were not used for the RT-qPCR and RNAseq experiments.

### 2.14 RNAseq experiments and analysis

hMφ-R were incubated for 6h with 100 nM TRB or LUR and RNA was extracted as indicated above. The RNAseq analysis was outscored by BGI. Briefly, Strand-Specific Transcriptome Library Construction Protocol (DNBSEQ) was used to determine whether a transcript comes from sense strand or antisense strand, and to identify the boundary of the transcript and more precisely the number of transcripts. This provided an important approach for gene fine structure and gene expression regulation. Strand-specific transcriptome library construction was completed by enriching mRNA from total RNA, sequenced by DNBSEQ high-throughput platform, and followed by bioinformatics analysis. mRNA molecules were purified from total RNA using oligo(dT)-attached magnetic beads and fragmented under controlled time and temperature. First Strand cDNA synthesis was achieved using the appropriate amount of primers by PCR. Second Strand cDNA synthesis was done by PCR using dUTP instead of dTTP. The reaction product was purified by magnetic beads. End repair and addition of A nucleotides were run by PCR under the action of enzymes, repairing the sticky ends of the cDNA double-stranded and adding A nucleotides to the 3'-end. Adaptor Ligation was obtained using a linker connection reaction system and PCR. The reaction product was purified by magnetic beads. Library quality control was validated on the Agilent Technologies 2100 bioanalyzer. The double-stranded PCR products were heat-denatured and circularized by the splint oligo sequence. The single-strand circle DNA (ssCir DNA) was formatted as the final library, amplified with phi29 to make DNA nanoball (DNB) which had more than 300 copies of one molecular, and the DNBs were loaded into the patterned nanoarray and single end 50 (pair-end 100/150) bases reads were generated in the way of combinatorial Probe-Anchor Synthesis (cPAS).

### 2.15 Gene expression analysis

RNA sequencing was carried out in the DNBseq^TM^ platform (Eukaryotic Strand Specific Transcriptome Resequencing product) applying its software to build the library (BGI; https://www.bgi.com/global/home). Data were deposited in the NCBI platform (GSE235390). On average, 50.9 M clean reads were generated. Data quality Q20 parameter=97.08%. Gene expression levels were calculated by the RSEM software package (28). Differential gene expression was filtered by DESeq2 algorithms (R-package) the parameters that were used to identify a gene as a DEG were the following: log2FC≥|1| and p<0.05. Ggplot2 package was used to elaborate plots and Genesis software (29) was the bioinformatic tool that allowed clustering and heatmap representation (http://genome.tugraz.at/genesisclient/). DEG enrichment sets were determined by ENRICHR (30–32) (http://amp.pharm.mssm.edu/Enrichr/), and statistical significance was calculated by a Benjamini-Hochberg test. To perform the gene set enrichment analysis (GSEA) (http://software.broadinstitute.org/gsea/index.jsp; (31)) broad Institute Data Base and NCBI Database were used (https://www.ncbi.nlm.nih.gov/gds). Functional annotation was conducted by consulting several databases: pathfindR (33), KEGG (https://www.genome.jp/kegg/)

The analysis was performed using the statistical computing environment R (4.1.1) in conjunction with the following packages: ComplexHeatmap (2.8.0) (34); EnhancedVolcano (1.10.0) (35) https://github.com/kevinblighe/EnhancedVolcano, gplots (3.1.1), ggplot2 (3.3.5) to create volcano plots, heatmaps and bubble charts; dplyr (1.0.7) to enable the dataset aggregation and analysis; VennDiagram (1.6.20) to create Venn diagrams graphic; and pathfindR (1.6.2) to perform enrichment analyses that identify active protein-protein interactions networks, identifying clusters of enriched terms and distinguish representative terms in each cluster. Package R was used for the statistical analysis of this section.

### 2.16 Quantification of cytokines and chemokines in cell culture supernatants

Human cytokines were determined in hMφ-R cell culture supernatants using LEGENDplex™ Human Essential Immune Response Panel (Biolegend, 740930) following the manufacturer’s instructions. Human macrophages were seeded at 2x10^6^ cells/well and were cultured with 1 mL of RPMI + P/S + 2% FBS for these experiments. Supernatants from vehicle, TRB or LUR 24h were collected and assessed.

### 2.17 Construction of condition-specific GSMMs

The generic human Genome-Scale Metabolic Model (GSMM) Recon3D (19) was used as a template for reconstructing the GSMMs of human macrophages under control conditions and different treatments (specific models for both TRB and LUR treatments). Computational analyses were performed using Python and the COBRApy toolbox (36–38).Recon3D provides a mathematical representation of the complete set of known metabolic reactions for *homo sapiens*, in a cell and tissue-agnostic manner. Recon3D, together with transcriptomics, respiration data, and medium constraints (RPMI and 2% FBS), was used as a base model for the reconstruction of condition-specific GSMMs. To simulate the metabolic, energetic, and reductive demands of macrophages, we implemented the macrophage biomass reaction previously described (39). In addition, to build condition-specific GSMMs, enzymes with fragments per kb of exon per million mapped fragments (FPKM) under 1 in all conditions were removed provided that their removal still enabled the models to produce 20% of optimal biomass as well to achieve the measured rates of respiration parameters. Additionally, differentially expressed (FDR< 0.05) enzymes with low expression in a drug-exposed condition but not in untreated samples were also removed from the model-specific to such condition.

### 2.18 Drug modulation of flux distribution using a quadratic metabolic transformation algorithm

First, the GIME3 algorithm (21) is applied to compute a reference flux distribution for the control condition. Briefly, this algorithm consists of a flux minimization weighted by gene expression subject to producing 20% of the optimal biomass production and matching the measured rates of respiration parameters. Next, flux variability analysis (19, 37) is used to identify the solution space within 99% of the GIME3 optimal solution. Finally, the resulting solution space is sampled using the Artificially Centered hit-and-run (ACHR) algorithm COBRApy (36, 38). The mean for resulting flux values defines the control/reference flux distribution.


$$\begin{array}{c} \min\;\sum_{m\;\in \;DExp}^{\;}\left(W_{m}\sum_{i\;\in \;R_{m}}^{\;}{\left(\frac{v_{i}^{ref}\cdot FC_{m}-v_{i}^{MTA}}{v_{i}^{ref}\left(FC_{m}-1\right)}\right)}^{2}\right)\\ +\sum_{i\;\in \;Ru}^{\;}\frac{{\left(v_{i}^{ref}-v_{i}^{MTA}\right)}^{2}}{v_{i}^{ref}}+\;\sum_{j\;\in \;Rexp}^{\;}\frac{{\left(E_{j}-v_{j}^{MTA}\right)}^{2}}{\sigma_{j}}\\ subject\;to:\\ s\cdot v_{\;}^{MTA}=0\;;\;\;\;lb<v_{\;}^{MTA}<ub\; \end{array}$$

**Formula 1.** Optimization performed by qMTA. *DExp*: set of differentially expressed genes *W_m_*: weight is given to gene *m R_m_*: reactions mapped to gene *m* (defined using Recon3D’s gene reactions rules); $v_{i}^{ref}$ and $v_{i}^{MTA}$ : control/reference and treatment fluxes, respectively; *FC_m_*: fold change of gene *m* expression relative to control; *Ru*: set of reactions not associated with differentially expressed genes or experimental measures; *Rexp*: set of fluxes measured experimentally *E_j_*: *s* experimental measure for reaction *j*; *σ_j_*: experimental standard deviation for reaction *j*; *s*: stoichiometric matrix of the condition-specific model; *lb* and *ub*: flux lower and upper bounds, respectively.

Next, qMTA (20) was applied to simulate the study of a transition from the control to each treatment as a result of the adaptation to the drug challenge (Formula 1). The optimization minimizes the difference between the simulated flux values and target flux (i.e. the product of the reference flux value by the gene expression fold change) for each differentially expressed gene mapped to any given reaction. For reactions that are not mapped to differentially expressed genes, the flux variation is minimized instead. Both terms of the optimization are scaled by the reference flux distribution to prevent a bias of reactions with high referenced flux. Additionally, for experimentally measured fluxes the difference between simulated and measured values is minimized and weighted by the experimental standard deviations.

Each differentially expressed gene is given a weight (Formula 2) defined by the adjusted p-value (FDR) for the fold change relative to the control.


$$W_{m}=LOG10\left(p_{th}\right)-LOG10\left(p_{m}\right)$$

**Formula 2.** Gene weight is based on their differential expression significance. *p_th_*: p-value threshold (0.25 FDR-adjusted p-value), which determines whether a gene is differentially expressed or; *p_m_*: FDR associated a given gene expression fold change.

### 2.19 Experimental data used for in-silico simulations

The model was trained with transcriptomic data from hMφ-R obtained from 4 healthy donors in the case of TRB (100 nM) and 5 for LUR (100 nM), being 10^7^ hMφ-R cultured per sample (RPMI + 2% FBS). Additionally, respiration parameters (OCR, basal respiration, and ATP production) from hMφ-R obtained from 2 was used as a model constraint, and hMφ-R were treated with either TRB (50 nM) or LUR (50 nM).

### 2.20 Lactate determination

Cell culture supernatants were collected after treatment with both drugs. Supernatants were used for the quantitative determination of lactate by enzymatic colorimetric assay (kit 1001330 Spinreact, Gerona, Spain) following the manufacturer’s instructions. Basal lactate contained in RPMI + 2% FBS was subtracted from the hMφ-R supernatants. hMφ-R control supernatants ranged from 4-8 mM and results were expressed in relative percentages.

### 2.21 Metabolites’ concentration determination

The concentration of intracellular content of a family of up to 180 metabolites, including amino acids, biogenic amines, acylcarnitines, lysophosphatidylcholines, phosphatidylcholines, sphingolipids and hexoses, were determined using the Absolute IDQ p180 kit from Biocrates Life Sciences (Innsbruck, Austria). The quantification was performed using an AB Sciex 4000 QTRAP MS/MS mass spectrometer coupled to an Agilent HPLC 1200, and following the next procedure: cell pellets containing 12x10^6^ hMφ-R cells, were resuspended in 70 μL of EtOH:PBS 85:15. Suspensions were treated twice as follows: suspensions were sonicated using titanium probe (3 x 15 seconds; output 25, tune 50), then submerged in liquid N_2_ for 30 seconds and thawed at 95°C in a dry bath. Suspensions were then centrifuged at 20,000*g* for 5 min at 4°C, and supernatants were collected and plated in the kit (10-30 μL for hMφ-R). Both kits containing media samples or cell pellets were processed following manufacturer’s instructions, and Analyst and the MetIDQ™ software packages were used to analyze the obtained data and calculate metabolite concentrations. Additionally, metabolite concentrations were determined spectrophotometrically using NAD(P)H-coupled enzymatic reactions in an autoanalyzer Cobas Mira Plus (Horiba ABX, Kyoto, Japan). Intracellular concentrations in cell pellets were corrected by protein content in cell lysates, measured using Bicinchoninic acid (BCA) assay.

### 2.22 Glucose/Galactose experiments

These experiments were carried out in DMEM +1%P/S+2%FBS. This medium does not contain glucose, galactose, pyruvate, glutamine or red phenol. 25 mM D-glucose or D-galactose (G5388, Sigma) were added extemporaneously. Experiments with hMφ-R were exposed to vehicle, 5 nM and 50 nM TRB or LUR to evaluate mitochondrial fitness and OXPHOS function (40).

### 2.23 Transmission electron microscopy

After 24h treatment with vehicle, TRB or LUR, cells were washed three times with sterile PBS, and subsequently fixed with Permanox®, in a chamber slide with a mix that contained 4% formaldehyde, 2% glutaraldehyde in cacodylate buffer, for 1h. After washing with the fixing solution, samples were fixed with 1% osmium tetroxide for 1h. Samples were contrasted with 0.5% aqueous uranyl acetate (pre-embedding). Subsequently, samples were dehydrated by adding increasing ethanol concentrations (30, 50,70, 95, 100%) and were gradually included in epoxy resin, to do so, samples were exposed to these solutions; ethanol-epoxy resin (2:1, 1:1, 1:2). To polymerize the epoxy resin, samples are incubated at 60°C for two days, once the epoxy resin is polymerized and dry, 60 nm ultrathin slides are cut with Leica ultramicrotome, samples are located in the grid. Finally, the grid and samples are treated with 2% uranyl acetate and Reynolds’ lead (post-embedding contrast). Ultrastructural studies were conducted using a Transmission Electron Microscope Jeol Jem 1010 equipped with a Gatan Orius200 SC digital camera with an acquisition tension of 80 kV.

### 2.24 Statistical analysis

Values in graphs correspond to mean ± SD. The statistical significance of differences between the means were determined with GraphPad Prism 9.0.0. (GraphPad Software) using a one-way analysis of variance (ANOVA) followed by Bonferroni post hoc test or Student’s t-test, as appropriate. A P-value< 0.05 was considered to be significant.

## 3 Results

### 3.1 TRB and LUR affect hMφ viability and induce ROS production and changes in the mitochondrial inner membrane potential

**Figure 1:**
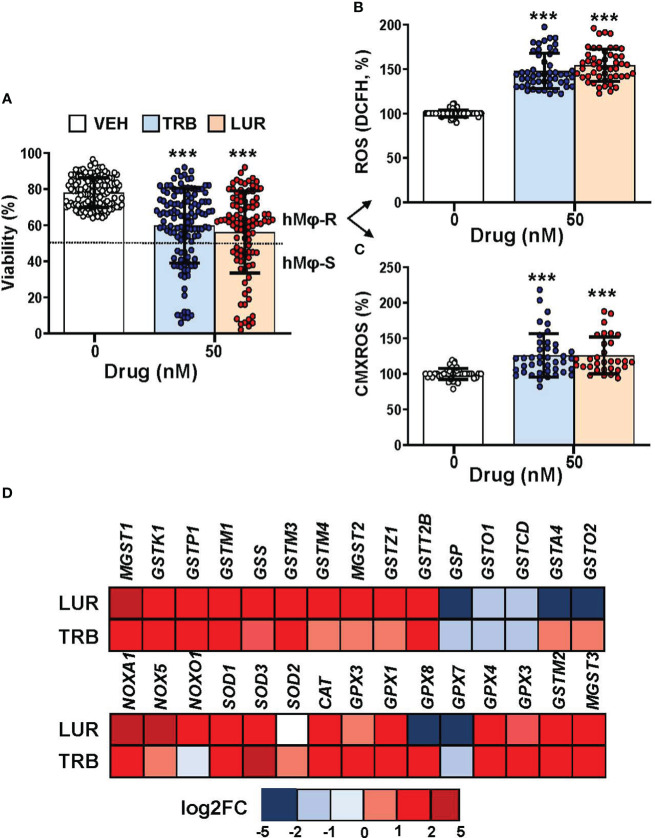
hMφ viability distribution, ROS production and mitochondrial inner membrane potential after 24h of treatment with 50 nM TRB or LUR. **(A)** Results show the mean ± SD of cell viability after treatment with TRB (n=130) or LUR (n= 100); **(B)** ROS production by hMφ-R after incubation with TRB or LUR (n=50); **(C)** Relative CMXROS fluorescence (in %) of hMφ-R after treatment with TRB (n=41) or LUR (n= 31). **(D)** Mean log2 of fold induction (F.I.) of genes involved in ROS production after treatment of hMφ-R (2x10^6^ cells) for 6h with TRB or LUR (n=5). ***p<0.001 *vs*. 0 hours with vehicle.

Recently, we reported that when hMφ from healthy blood donors were treated for 24h with TRB or LUR up to 100 nM (in the therapeutic range) there were differential responses in terms of cell viability (15), indicating a stratification regarding the overall effect of these drugs. This observation could potentially have an impact on the outcome of patients treated with these anti-tumor drugs. **Figure 1A** shows the hMφ viability distribution of 130 and 100 healthy blood donors treated with TRB and LUR, respectively. After establishing a cut-off of 50% loss in viability, two subgroups can be identified: A resistant group (hMφ-R) that accounts for 65% of the population that is barely affected by the treatment with these drugs (**Supplementary Figure S1**), and a sensitive group (hMφ-S) constituted by 35% of the population in which cell viability dramatically halves in the presence of TRB or LUR. A statistically significant increase in ROS production (**Figure 1B**) and mitochondrial inner membrane hyperpolarization (**Figure 1C**) were observed after incubation of hMφ-R with these drugs. The increase in ROS production was associated with the overexpression of genes coding for glutathione-related enzymes (**Figure 1D**).

**Figure 2:**
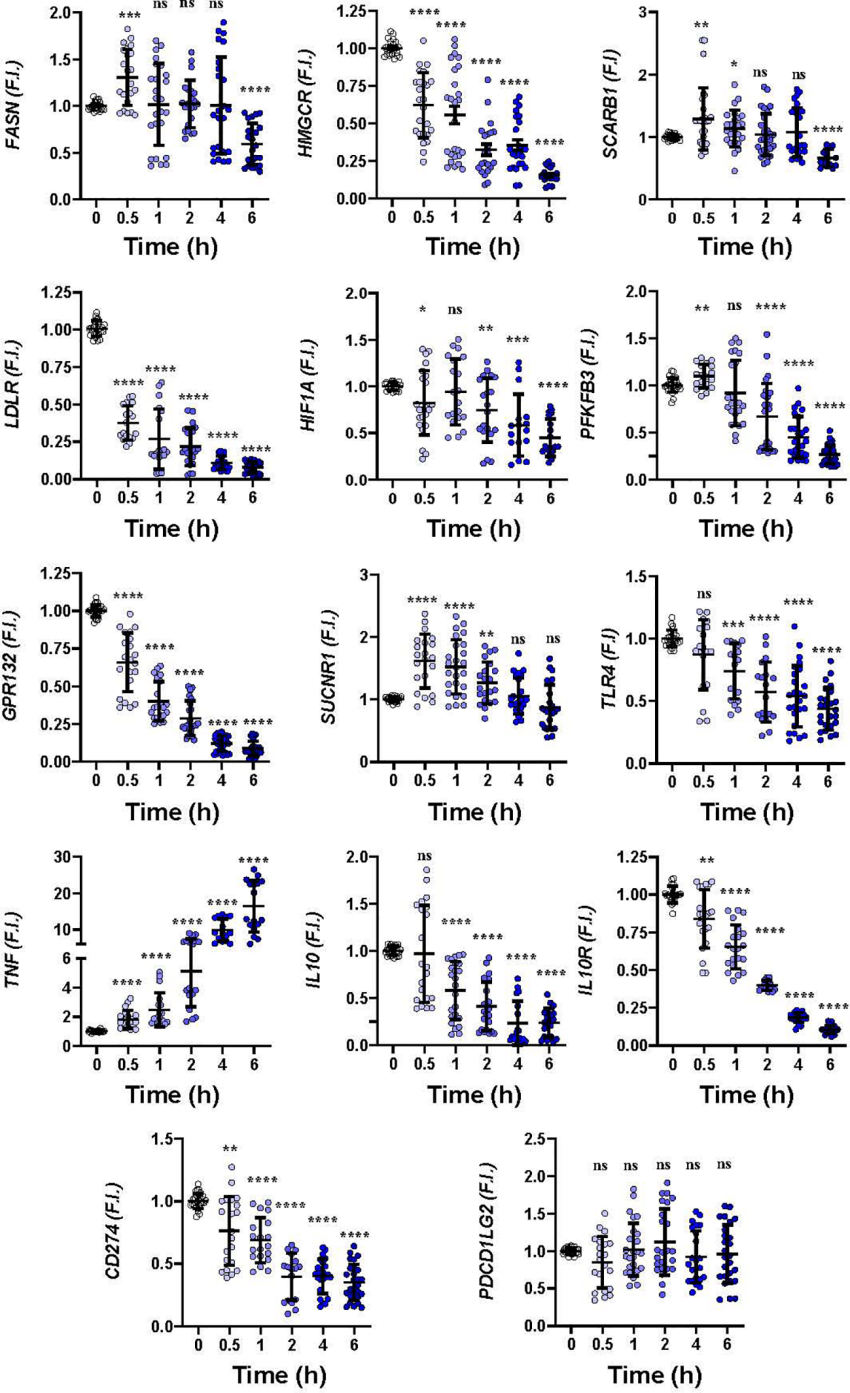
Gene expression time course patterns in hMφ-R after treatment with 100 nM TRB. hMφ-R-RNAs from healthy donors were isolated and underwent real-time RT-qPCR using the indicated primers for each gene. Results show the mean ± SD of fold induction (F.I.) from 10 different donors (10^7^ cells, each) assayed per triplicate. *p<0.05; **p<0.01; ***p<0.001; ****p<0.0001 *vs*. 0 hours with vehicle. ns, not statistically significant.

To gain insight into how hMφ-R respond to these drugs a time-course analysis of the mRNA levels of relevant genes in hMφ-R polarization was performed. As **Figure 2** shows, significant transcriptional repression was observed for lipid and cholesterol biosynthesis-related mRNAs, such as *FASN, HMGCR*; similarly, mRNAs that codify LDL-related receptors: *LDLR, GPR132*, and glycolysis regulation genes, such as *HIF1A* and *PFKFB3* were downregulated. In addition, there was transcriptional repression of anti-inflammatory genes, such as *IL10R* and *IL10*, and *CD274* that encodes for PDL1, a receptor that binds to PD1 and induces T-cell exhaustion and/or anergy in lymphocytes (41). A transient increment in *SUCNR1*, and a robust time-dependent expression of *TNF* were observed. As **Supplementary Figure S2** shows, similar results were obtained when hMφ-R were treated with LUR, which suggests that hMφ-R are orchestrating a common transcriptional regulation program in response to TRB and LUR.

### 3.2 TRB and LUR trigger the activation of pro-inflammatory pathways in hMφ-R

**Figure 3:**
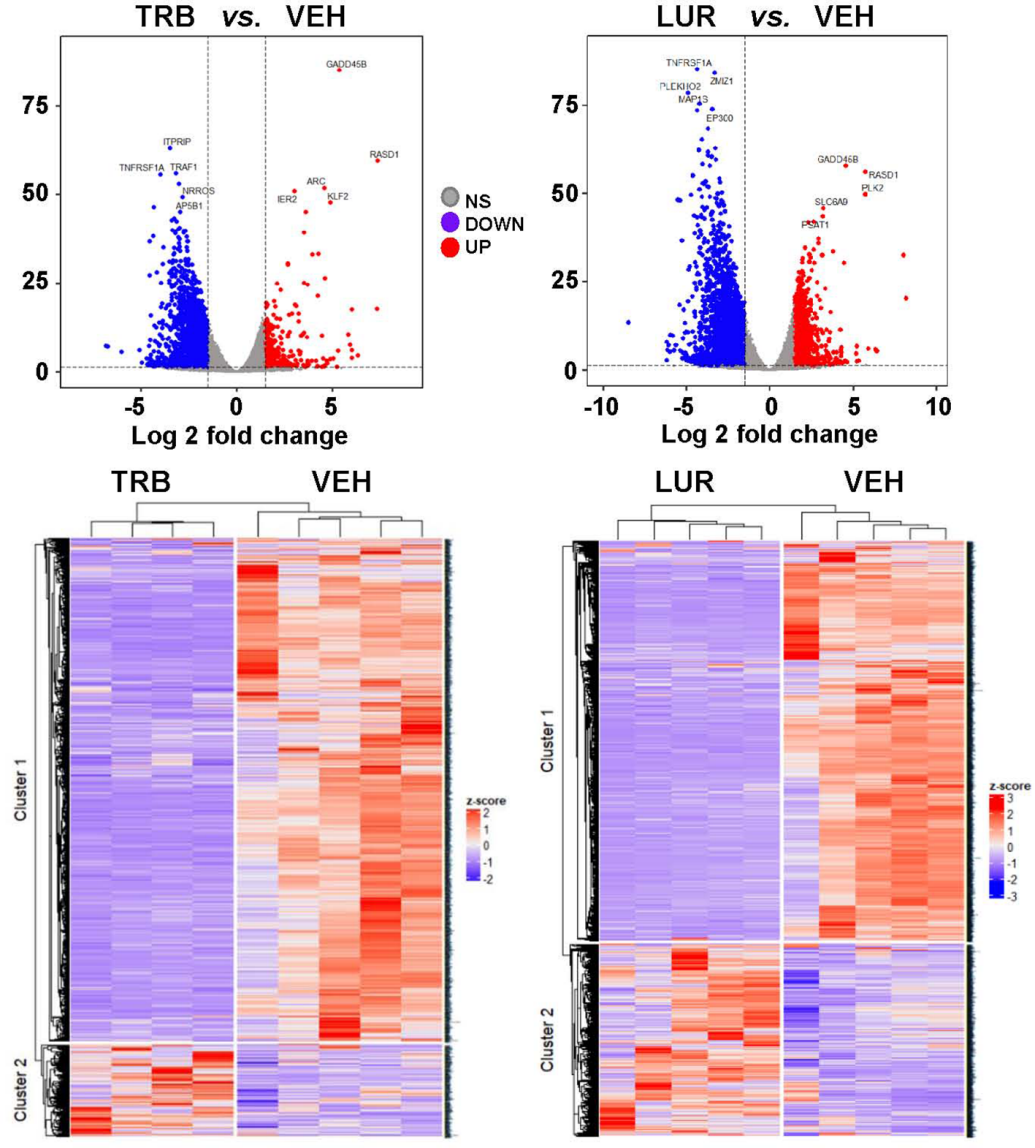
hMφ-R RNAseq gene expression pattern array after treatment for 6 hours with 100 nM TRB or LUR. Results show the mean log2 of fold induction (F.I.) from 5 different donors (10^7^ cells, each) *vs*. 6 hours with vehicle.

**Figure 4:**
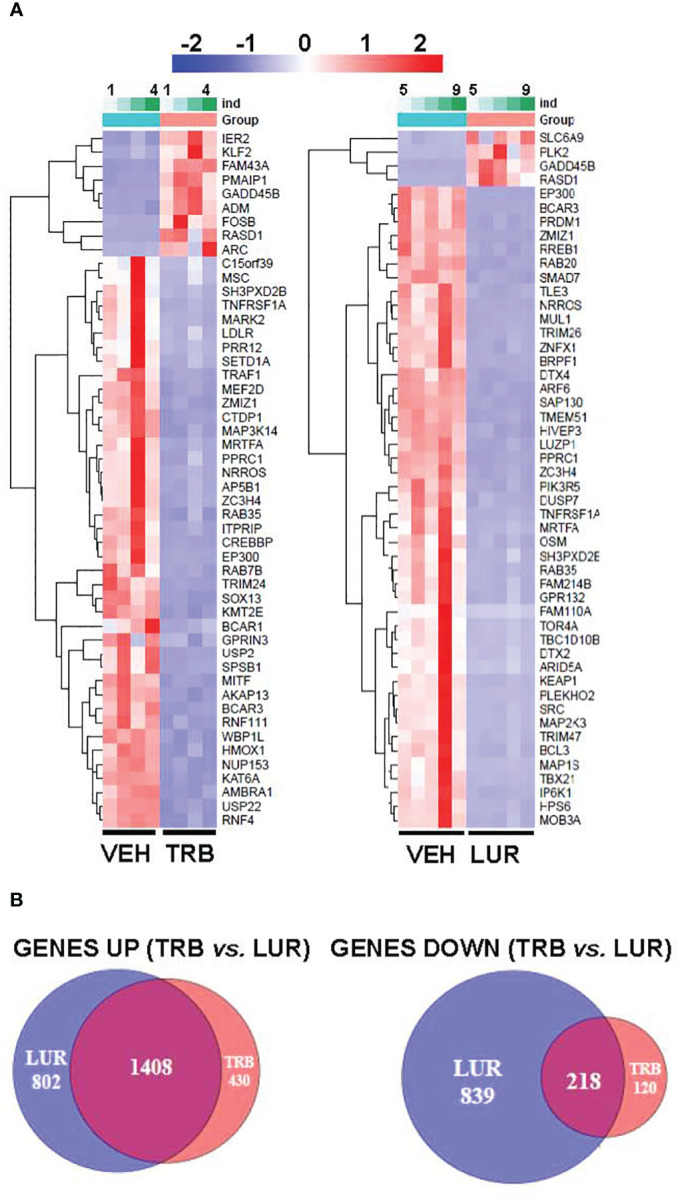
RNAseq identification of top 50 differentially expressed genes **(A)** and overlapping transcriptome **(B)** of hMφ-R after 6 hours of treatment with 100 nM TRB or LUR. Results show the mean log2 of fold induction (F.I.) from 5 different donors (10^7^ cells, each) *vs*. 6 hours with vehicle.

Analysis of the transcriptional regulation induced by TRB and LUR showed similar patterns, prevailing a downregulation of mRNAs (**Figure 3**). This repression accounts for 52% of the differentially expressed genes (DEGs) (7,490/13,824 genes) in TRB and 54% in LUR (3,770/7,020 genes). Furthermore, there was 32% overlap (12/50, **Figure 4A**) of the top 50 DEGs induced by each drug. *GADD45B* and *RASD1* were the only common overexpressed genes in TRB and LUR treated hMφ-R. Regarding the upregulated genes in response to TRB, *IER2* and *KFL2* codify for transcription factors that positively regulate the JNK/p38 inflammatory pathway. Among the highest DEG in response to TRB were *PMAIP1* (PMA Induced Protein 1), which favors apoptosis and induces changes in the mitochondrial membrane efflux (42), and *RASD1*, a small Ro-GTPase. A similar analysis in the presence of LUR showed overexpression of *SLC6A9*, which codifies for the glycine solute carrier 1 (GlyT1) and *PLK2*, which codifies for a protein that participates in normal cell division, specifically, in centriole duplication in mammalian cells (43). However, macrophage proliferation is very restricted (i.e., within the atheroma plaque). These upregulations may be explained by genotoxic stress. *PKL2* activates antiviral innate immune cell responses (44), and *IER2* is an early response gene involved in cell adhesion and motility (45, 46). TRB and LUR induce extensive transcriptional repression; both molecules downregulate *SH3PXD2B*, which is implicated in macrophage migration (47) and could partially explain hMφ-R phagocytosis impairment (15). Also, these compounds inhibit cytoskeleton dynamics by repressing *MRTFA*, *SRF*, *RAB35* and *BCAR3* mRNAs. This observation is supported by previous data (48) demonstrating that LUR strongly inhibited Rho GTPase family gene expression and impaired human monocyte migration.

To show the overlapping transcriptome between TRB and LUR in hMφ-R a Venn diagram was used (**Figure 4B**). An overlap exists of 76.6% of the overexpressed genes in the case of TRB (1,408 *vs.* 1,838 in total) which are simultaneously overexpressed in LUR and that accounts for 63.3% (1,408 *vs.* 2,210 in total) within this subset, leaving a differential of 430 genes that are uniquely overexpressed by TRB (23.4%) and 802 genes (37.6%) that are upregulated by LUR. Hence, the overlapping in the upregulated genes is high, and LUR induces more changes than TRB at the transcriptional level. Conversely, there is less overlapping in the downregulated gene expression subset for TRB; it accounts for 64.5% (218 *vs.* 318) which are also downregulated in LUR, but this overlap only constitutes the 20.8% of downregulated genes in LUR; 79.2% (839/1047) are only downregulated in LUR. This fact suggests that LUR exerts a higher transcriptomic impact in hMφ-R and potentially regulates more molecular mechanisms and biological functions in these cells.

Functional *in silico* studies using gene ontology (GO) term annotation for hMφ-R treated with TRB or LUR showed that both compounds positively modulate the expression of MHC class I protein complexes, antigen processing and peptide antigen presentation (MHCII), ribosome-related transcripts, and serine, glycine and threonine metabolism (**Supplementary Figure 3**). Other molecular GO terms are negatively regulated: DNA repair, covalent chromatin modification and histone modifications. There are additional signaling mechanisms that involve the immune system and are predicted to be inhibited; within this category, it is relevant to mention the small GTPases transduction. Different databases were used to perform a systematic study of the molecular pathways that are affected by these drugs. According to GO terms for functional annotation of biological processes (pathfindR; **Supplementary Figure 4A**) TRB and LUR positively regulated NF-κB. Additionally for TRB, regulation of small GTPases, and JNK signaling pathways are transcriptionally upregulated, whereas the regulation triggered by LUR impacts protein phosphorylation, which is consistent with the calcium signaling responses previously described (15) as well as RNA transcription factor activity modifications induced by this drug. The same bioinformatics approach was followed using KEGG database. TRB specifically triggers ubiquitin-mediated proteolysis, ErbB and T-cell receptor pathways. In contrast, LUR triggers lysosome trafficking, pathogenic *E. coli* infection and NF-κB signaling pathways. Graphical details of these pathfindR and KEGG pathways are given in **Supplementary Figures S5A, B**. These data indicate that TRB and LUR induce an acute pro-inflammatory activation in hMφ-R which could explain, at least in part, a functional mechanism that boosts the antitumor activity of innate immune cells and counteracts the immunosuppressive microenvironment established by tumors and their stromal supporting cells. **Supplementary Figure S6** recapitulates the molecular functions in gene set enrichment analyses (GSEAs) that are altered by TRB and LUR.

Metabolically, hMφ-R are essentially dependent on glucose for homeostasis and pro-inflammatory activation (3). To investigate how TRB and LUR are modulating hMφ-R immuno-metabolism, cell culture supernatants were collected after treatment with both drugs and lactate production was measured as read out of glycolytic activity (**Supplementary Figure S7A**). To elucidate how TRB modulates metabolic profile, Absolute*IDQ* p180 kit was used to analyze the intracellular content of amino acids (**Supplementary Figure S7B**). There were statistically significant changes in the content of five amino acids: aspartate, glutamine, histidine, proline and in t4-OH-proline whose cell content was decreased when cells were treated with TRB.

### 3.3 TRB and LUR impact mitochondrial biogenesis and function in hMφ-R

**Figure 5:**
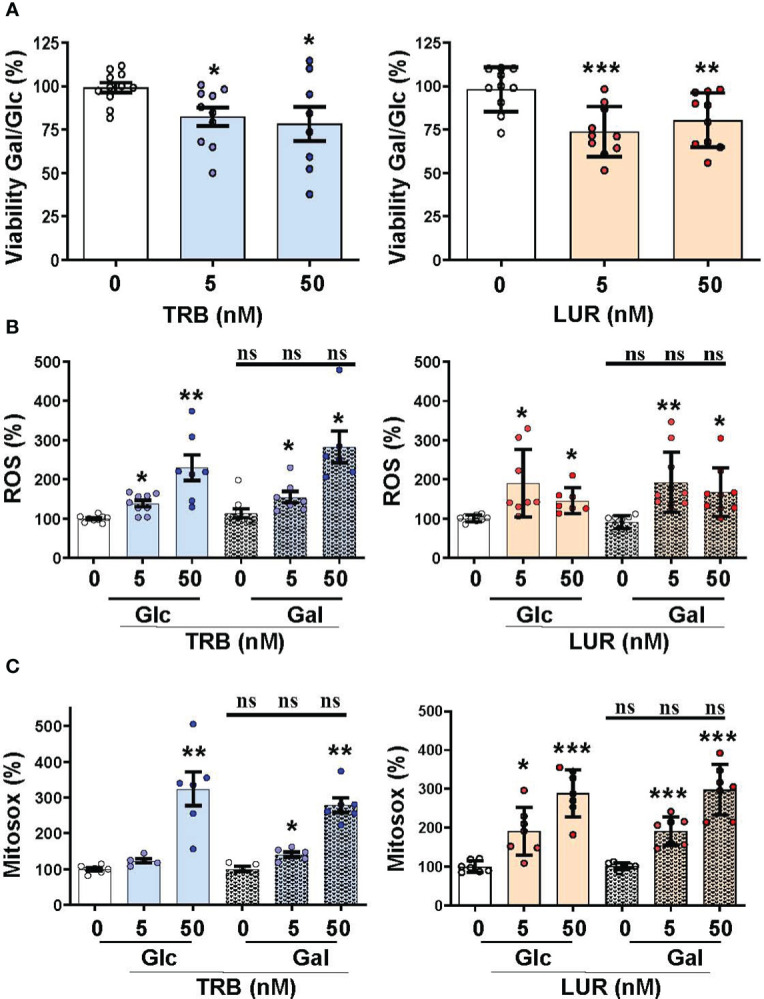
Mitochondrial function impairment in hMφ-R maintained in 25 mM glucose (Glc) or galactose (Gal) and in the presence of TRB or LUR. hMφ-R were incubated in RPMI1640 medium and Glc or Gal as hexose substrate. Cells (2x10^6^) were treated for 24h with the indicated concentrations of TRB and LUR. **(A)** Cell viability after incubation with galactose (Gal) or glucose (Glc); **(B)** ROS production; **(C)** mitochondrial ROS production. Results show the mean ± SD of the indicated parameters (expressed in %) from 9 different healthy donors. *p<0.05; **p<0.01; ***p<0.001 *vs*. the vehicle condition. ns, not statistically significant.

We previously reported that TRB and LUR inhibited oxygen consumption in hMφ-R (15). To evaluate the relative dependence of glycolytic *vs*. mitochondrial ATP synthesis hMφ-R were cultured for 24h with 25 mM glucose (glycolytic ATP production) or 25 mM galactose (no ATP is produced by glycolysis) (**Figure 5**). hMφ-R were significantly more sensitive to 50 nM TRB and LUR in 25 mM galactose *vs*. their counterparts in glucose (**Figure 5A**). This fact was corroborated by conducting the same experiments at 72h (**Supplementary Figure S8**). Quantification of ROS production from the cytoplasm or the mitochondria showed a statistically significant rise after TRB or LUR treatment; however, these effects were independent of the hexose source (**Figures 5B, C**).

**Figure 6:**
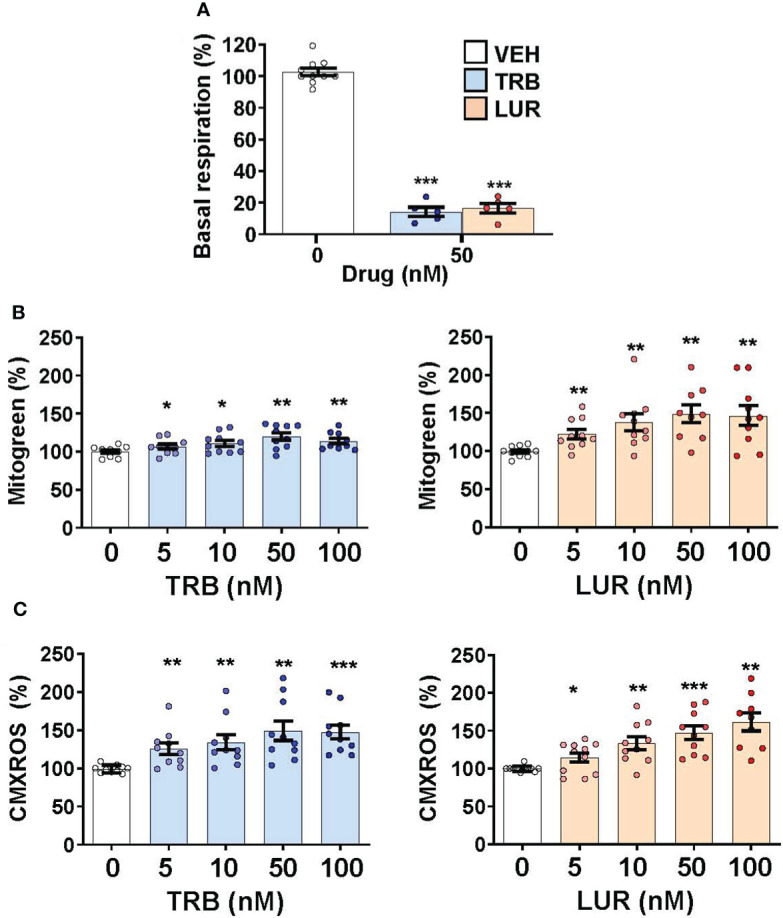
Treatment of hMφ-R with TRB or LUR favors mitochondrial biogenesis and membrane hyperpolarization. hMφ-R (2x10^6^ cells) were analyzed after 24h of treatment with TRB or LUR. **(A)** Basal respiration; **(B)** mitochondrial mass evaluated with Mitogreen; **(C)** mitochondrial inner membrane potential evaluated with CMXROS. Results show the mean ± SD from 10 different healthy donors. *p<0.05; **p<0.01; ***p<0.001 *vs*. the vehicle condition.

Since hMφ-R treated with TRB or LUR reduced their oxygen consumption rate but preserved cell viability (**Figures 1A**, **6A**) we hypothesized that there might be a compensatory biosynthetic mechanism supporting energy demands. Indeed, hMφ-R were treated with TRB and LUR and incubated with mitogreen, an indicator for mitochondrial mass. Mitogreen incorporation significantly raised, suggesting the occurrence of mitochondrial biogenesis (**Figure 6B**) and, concomitantly, there was an increase in CMXROS fluorescence (an indicator of the mitochondrial inner membrane potential; **Figure 6C**). These data were supported at the transcriptomic level, which showed an increase in the transcripts that encode for OXPHOS complexes within the nuclear genome; there was an overall overexpression for nearly 100 mRNAs shown in **Supplementary Figure S9**.

**Figure 7:**
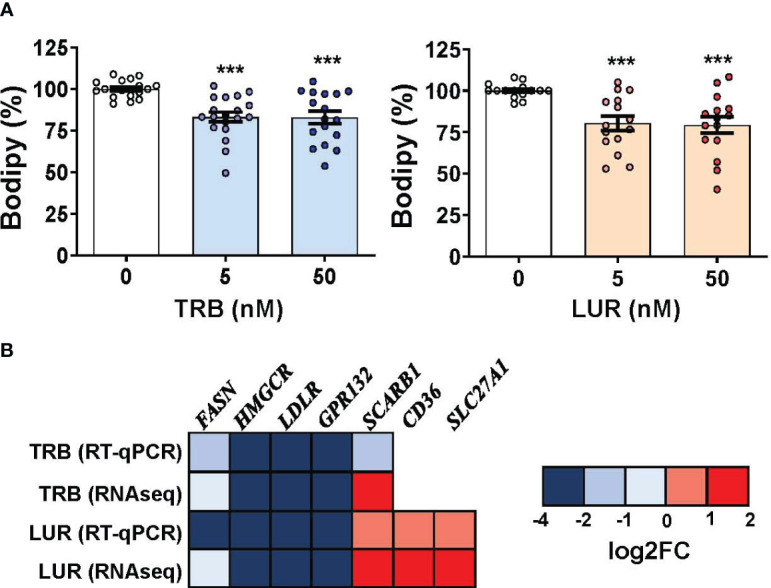
TRB and LUR induce a reduction in neutral lipid content and a transcriptional repression in lipid-metabolism-related enzymes in hMφ-R. Cells (2x10^6^) were analyzed after 24h of treatment with TRB or LUR. **(A)** Bodipy incorporation; **(B)** selected expression profile of lipid-metabolism genes. Results show the mean ± SD from 19 (TRB) and 16 (LUR) different healthy donors, and 6 donors for RT-qPCR/RNAseq (at 6h with 100 nM TRB or LUR). ***p<0.001 *vs*. the vehicle condition.

Because TRB and LUR induced significant transcriptional repression in scavenger receptors (*LDLR* and *GPR132*) and essential anabolic enzymes that play a role in lipid and cholesterol metabolism (*FASN* and *HMGCR*) we evaluated the neutral lipid content in hMφ-R challenged with TRB or LUR. As **Figure 7A** shows, Bodipy incorporation decreased *ca.* 20-25% when exposed to TRB or LUR. In contrast with these observations, other fatty acid receptors are overexpressed such as *CD36* and *SLC27A1* (**Figure 7B**). These data suggest changes in membrane fluidity and hMφ-R function, as well as activation of lipid catabolic processes as RNAseq predicts for TRB and LUR (**Supplementary Figure S3**).

Since changes in the physical properties of mitochondria and neutral lipid content were observed, we performed transmission electron microscopy experiments (**Supplementary Figure S10**). At 40 kx LUR promotes leakage of the cytoplasmic compartment, and consequently, a reduction in the negative stain of macrophages. The black dots are surrounded by a single membrane, mainly after LUR treatment, these structures look like azurophilic granules (referred to as tertiary granules in the RNAseq data, **Supplementary Figure S3**), and probably accumulate hydrolytic hydrolases and peroxidases, secondary lysosomes or cargos of processed autophagosomes. There were no evident changes in the ultrastructural features of mitochondria. To confirm whether these drugs affected lysosome trafficking, lysotracker measures were obtained by flow cytometry, showing an increase of *ca.* 16-20% when challenged with TRB or LUR, respectively (**Supplementary Figure S11**).

**Figure 8:**
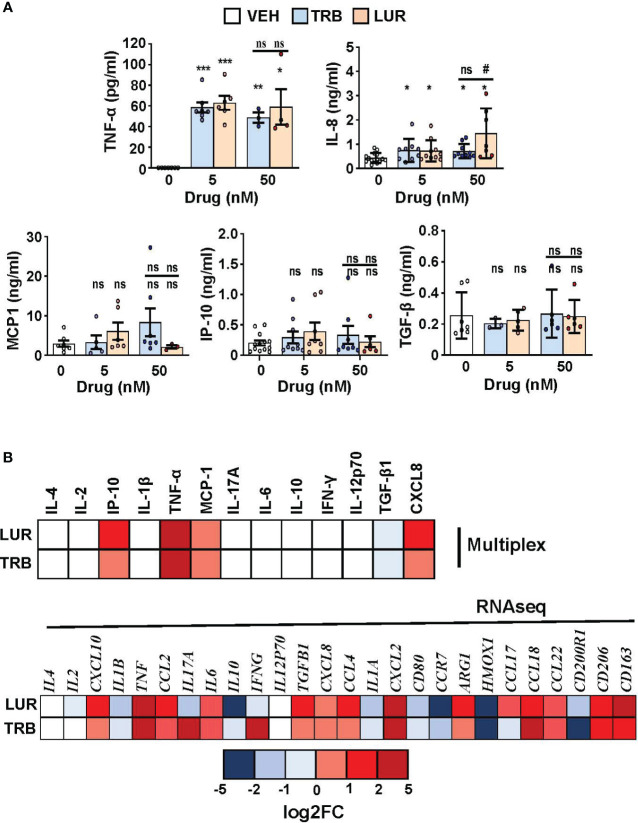
hMφ-R cytokine production profile and comparison with RNAseq data after incubation with TRB or LUR. **(A)** Supernatants from hMφ-R cells (2x10^6^) were analyzed after 24h of treatment with 5 nM and 50 nM TRB or LUR (n=7); **(B)** Cells were treated with 100 nM TRB or LUR for 6h in the RNAseq experiments. Results show the mean ± SD of the indicated cytokines. *p<0.05; **p<0.01; ***p<0.001 *vs*. the vehicle condition, #p<0.05 *vs*. the 5 nM condition. ns, not statistically significant.

Finally, a human multiplex inflammatory panel (LEGENDPLEX) was used to quantify the secretion of essential cytokines produced by hMφ-R in response to TRB or LUR treatment (**Figure 8A**). An accumulation of TNF-α (60 pg/mL) and IL-8 (726 pg/mL and 1450 pg/mL *vs*. 433 pg/mL, at 5 nM of the drug) was detected in response to the drugs. However, there were no changes for other relevant mediators. In addition, **Figure 8B** shows the concordance between RNAseq data and released protein levels into the supernatant measured by multiplex.

### 3.4 TRB and LUR increase glycolysis, pentose phosphate pathway, and TCA cycle fluxes

**Figure 9:**
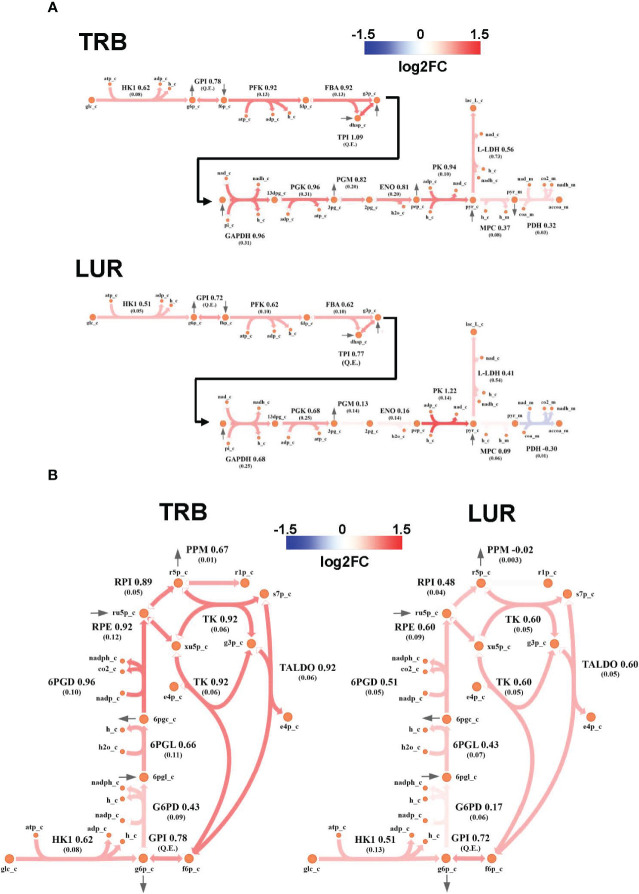
Metabolic flux map of TRB and LUR modulation of glycolysis and PPP in hMφ-R. **(A)** Flux upregulation and downregulation are shown in red and blue, respectively. Each reaction is presented as the enzyme/transporter ID, associated log2FC value of the flux, and the predicted flux value (parenthesis). HK1, hexokinase 1; GPI, glucosa-6-phosphate isomerase; PFK, phosphofructokinase; FBA, fructose-1,6-bisphosphate aldolase; triosephosphate isomerase; phosphoglycerate kinase; PGM, phosphoglycerate mutase; ENO, enolase; MPC, mitochondrial pyruvate carrier; PDH, pyruvate dehydrogenase; L-LDH, L-lactate dehydrogenase; glc, glucose; g6p, glucose-6-phosphate; f6p, fructose-6-phosphate; fdp, fructose 1,6-bisphosphate; dhap, dihydroxyacetone phosphate; g3p, glyceraldehyde-3-phosphate; 13dpg, 1,3-bisphosphoglycerate; 3pg, 3-phosphoglycerate; 2pg, 2-phosphoglycerate; pep, phosphoenolpyruvate; pyr, pyruvate; lac_L, L-lactate; accoa, acetyl-coenzyme A; atp, adenosine triphosphate; adp, adenosine diphosphate; h, proton; nad(h), nicotinamide adenine dinucleotide; nadp(h), nicotinamide adenine dinucleotide phosphate; pi, inorganic phosphate; coa, coenzyme-A; _c, cytosolic; _m, mitochondrial; Q.E., quasi-equilibrium. **(B)** PPP flux upregulation and downregulation are shown in red and blue, respectively. Each reaction is presented as the enzyme/transporter ID, associated log2FC value of the flux, and the predicted flux value (parenthesis). HK1, hexokinase 1; GPI, glucose-6-phosphate isomerase; G6PD, glucose-6-phosphate dehydrogenase; 6PGL, 6-phosphogluconolactonase; 6PGD, 6-phosphogluconate dehydrogenase; RPE, ribulose-phosphate 3-epimerase; RPI, ribose-5-phosphate isomerase; PPM, phosphopentomutase; TK, transketolase; TALDO, transaldolase; glc, glucose; g6p, glucose-6-phosphate; f6p, fructose-6-phosphate; 6pgl, 6-phosphogluconate; ru5p, ribulose-5-phosphate; r5p, ribose-5-phosphate; r1p, ribose-1-phosphate; xu5p, xilulose-5-phosphate; s7p, sedoheptulose-7-phosphate; e4p, erythrose-4-phosphate; g3p, glyceraldehyde-3-phosphate; h, proton; nad(h), nicotinamide adenine dinucleotide; nadp(h), nicotinamide adenine dinucleotide; _c, cytosolic.

**Figure 10:**
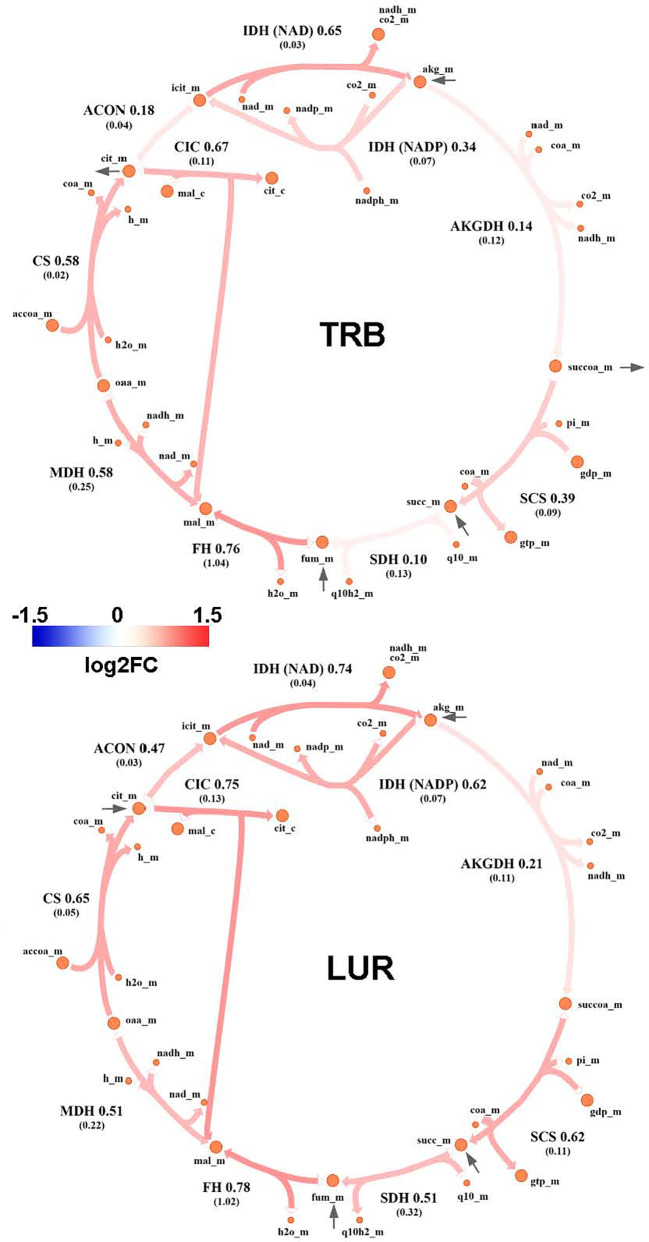
Metabolic flux map of TRB and LUR modulation of the TCA cycle in hMφ-R. Flux upregulation and downregulation are shown in red and blue, respectively. Each reaction is presented as the enzyme/transporter ID, associated log2FC value of the flux, and the predicted flux value (parenthesis). CS, citrate synthase; ACON, aconitase; IDH, isocitrate dehydrogenase; AKGDH, α-ketoglutarate dehydrogenase; SCS, succinate synthase; DIC, dicarboxylate carrier; SDH, succinate dehydrogenase; FH, fumarase; MDH, malate dehydrogenase; accoa, acetyl-coenzyme A; coa, coenzyme A; cit, citrate; icit, isocitrate; akg, α-ketoglutarate; succoa, succinyl-coenzyme A; succ, succinate; so3, sulphite; gtp, guanosine trisphosphate; gdp, guanosine diphosphate; pi, phosphate inorganic; fum, fumarate; mal, malate; oaa, oxaloacetate; h, proton; nad(h), nicotinamide adenine dinucleotide; nadp(h), nicotinamide adenine dinucleotide phosphate; fad(h_2_), flavin adenine dinucleotide; _c, cytosolic; _m, mitochondrial.

Genome-Scale Metabolic Modeling (19–21) methods indicated that treatment of hMφ-R with TRB or LUR is predicted to increase fluxes throughout the glycolysis and both the oxidative and non-oxidative branches of the PPP (**Figures 9A, B**). TRB displays large increases in fluxes through these pathways. Interestingly, this is especially relevant for the metabolic flux passing through the oxidative branch of the PPP, which is predicted to be less responsive upon LUR treatment. The fate of glucose-derived pyruvate is metabolized to lactate *via* L-LDH, rather than converted into acetyl-CoA *via* PDH for boosting the TCA cycle. Moreover, this pathway appears to be repressed in LUR-treated hMφ-R (log2FC_LUR_ = -0.30). Although the incorporation of pyruvate into the TCA cycle is less favored than lactate (**Figure 10**), every flux from the cycle is predicted to be upregulated in both TRB- and LUR-treated hMφ-R due to metabolite exchanges and transports across the mitochondrial membrane. The impact of LUR on these metabolic fluxes appears to be slightly stronger than TRB. The TCA cycle is impaired in two reactions: isocitrate dehydrogenase (NADP-dependent) and malate dehydrogenase (MDH). The model predicts that citrate is produced from both oxaloacetate and α-KG. Interestingly, the citrate overproduction leads to an increase in the citrate export into the cytoplasm in exchange for malate *via* CIC (log2FC_TRB_ = 0.67, log2FC_LUR_ = 0.75). The model predicts that malate is produced from both fumarate and oxaloacetate *via* fumarase and MDH, respectively. Furthermore, both reactions appear to exhibit increased activity following treatment with TRB and LUR. In addition, our results show that cytoplasmic/mitochondrial transports of α-KG and succinate (**Supplementary Table S1**) are highly modulated by both drugs, especially by LUR. Following TRB and LUR treatments, hMφ-R tend to export less succinate from the mitochondria into the cytoplasm (log2FC_TRB_ = -1.11, log2FC_LUR_ = -5.93), whereas they internalize more α-KG (log2FC_TRB_ = 1.25, log2FC_LUR_ = 2.07).

### 3.5 TRB and LUR trigger serine and methylglyoxal synthesis in human macrophages

A substantial fraction of glycolysis-derived 3-PGL is metabolized into 3PHP *via* PHGDH (**Supplementary Figure 12A**). Moreover, the impact of both drugs in this pathway is higher than in the corresponding glycolytic step (PHGDH: log2FC_TRB_ = 1.26, log2FC_LUR_ = 1.97 vs. PGM: log2FC_TRB_ = 0.82, log2FC_LUR_ = 0.13). Successive reactions from this cascade (PSAT and PSPH) are also upregulated by both drugs, leading to serine biosynthesis. Moreover, treatment of hMφ-R with TRB or LUR enhances the synthesis of methylglyoxal from hydroxyacetone (acetol), while it highly represses its generation from glyceraldehyde-3-phosphate/dihydroxyacetone-phosphate at the catalytic center of TPI (**Supplementary Figure 12B**). The last effect is stronger in the case of TRB, as it is predicted to inhibit glycolysis-derived methylglyoxal. Regarding methylglyoxal degradation *via* the glyoxalase system (GLO1, GLO2), incubation of hMφ-R with either TRB or LUR leads to increased methylglyoxal detoxification, thus resulting in the generation of D-lactate. Taken together, these results are consistent with an M1-like polarization state when hMφ-R are treated with TRB or LUR.

## 4 Discussion

We have compared the specific responses to each drug in hMφ in terms of cell viability, polarization and functional and metabolic responses since these cells play a key role in modulating essential biological functions including host defense against pathogens, wound healing, immuno-surveillance, cancer and autoimmune diseases (49, 50). These phagocytic professional cells regulate their transcriptomic signature to orchestrate specific effector functions and adapt to respond to a dynamic microenvironment. In this regard, it is relevant to understand the mechanism of action of these antitumor agents to develop stratified approaches for their optimal therapeutic use. Previously, we described the impact of these compounds in hMφ-S, which showed cell death upon incubation with TRB or LUR. These cells exhibit mitochondrial-associated caspase 9 activation and apoptosis (15). This fact, *per se*, could be of pivotal importance since hMφ could contribute to the promotion of tumor growth (tumor-associated macrophages) by inhibiting T-cell-mediated responses (51).

We identified a partial proinflammatory activation induced by both TRB and LUR. When hMφ-R are exposed to TRB or LUR cytoplasmic and mitochondrial ROS production rises, with a concomitant transcriptomic activation of the oxidative branch of PPP that is reflected in a catalase and superoxide dismutases overexpression. Thus, these compounds potentiate hMφ-R antitumor capacities by enhancing the oxidative PPP branch for the generation of reductive equivalents *via* NADPH oxidase that is required for ROS production. Regarding mitochondrial function, cell basal respiration is highly depressed upon TRB or LUR treatment; however, an early mitochondrial biogenesis transcriptional program occurs as a compensatory mechanism. This defect is corroborated by Leloir cycle experiments where it is shown that hMφ-R are more sensitive to both drugs in the presence of galactose.

Regarding the differential transcriptomic landscape of hMφ-R treated with TRB and LUR we observed similar transcription patterns when compared to naïve hMφ-R and DEG were commonly modulated. As relevant issues, it should be mentioned the overexpression of *GADD45*, coding for a protein related to DNA damage and the activation of P38/JNK inflammatory signaling pathway when the cell cycle is arrested (52). Regarding repressed genes, it is relevant to mention *SH3PXD2B*, which is implicated in macrophage and dendritic cell migration within tissues (47), probably contributing to the reduced phagocytic activity after drug incubation (15, 48); *NRROS*, which negatively regulates and limits ROS production during inflammatory responses and could explain the predicted activation of NF-κB and the rise in ROS species (53); *TNFRSF1A*, one of the most repressed DEGs (log_2_FC_TRB_= -4 and log_2_FC_LUR_ = -4.35, respectively), which codifies for a TNF receptor superfamily and is considered a death-receptor due to the presence of a death domain. It is related to cytotoxic signaling pathways, but it also allows the activation of inflammatory pathways such as NF-κB and MAPK (54); and *ZMIZ1*, which codifies for a protein inhibitor of activated STATs (55).

The associated GO terms for molecular pathway analysis predict the activation of acute inflammatory processes, humoral immune responses, Th-17 signaling pathways as well as positive regulation of cell death and inhibition of cell proliferation. For LUR, the predicted biological function activation involves antigen processing and presentation and lipid catabolic processes, lysosomal trafficking and tertiary granule formation.

These conclusions are supported by GSMM-based fluxomic studies; hMφ-R glycolysis is enhanced by both drugs and the TCA cycle presents the classical two breaks at the citrate accumulation and succinate oxidation level, which favors PPP, HIF1-α stabilization and ROS production mimicking a proinflammatory metabolic profile (56). Quantitative determination of intracellular metabolites in the Biocrates platform showed a significant decrease of glutamine, aspartate, histidine, proline and t4-OH proline levels in hMφ-R treated with TRB. At this point, it is relevant to mention that in cultured cells, all nutrients are over the physiological range, from carbon sources to amino acids. This is particularly relevant for glutamine since it has been reported that this amino acid significantly reduces the upper part of glycolysis interfering with the activation state of 6-phosphofructo-1-kinase (57).

The other arm of the metabolic flow as a whole is lipid metabolism. Normally, macrophages accumulate triglycerides (TG) in lipid droplets that are stained by Bodipy, in addition to other lipids from membranes. Treatment of hMφ-R with TRB or LUR reduces Bodipy staining by *ca*. 20%, which suggests that these lipids are catabolized as a consequence of drug treatment. Indeed, RNAseq data show a rise in the levels of *CD36, SCARB1* and *SLC27A1.* The upregulation of these fatty acids receptors/transporters suggests that hMφ-R are trying to internalize fatty acids to replenish the fatty acid pool. *SLC27A1* is overexpressed. It codifies for a long-chain fatty acid transporter protein that has been reported to enhance the macrophage inflammatory response by coupling ceramide and c-Jun N-terminal kinase signaling (58). Noteworthy, this fact reinforces the concept that hMφ-R are polarized to a pro-inflammatory functional state after TRB and LUR treatment.

In this mild pro-inflammatory scenario due to TRB and LUR treatment, multiplex analysis for the presence of inflammation-related mediators revealed that TNF-α and IL-8 were significantly produced by hMφ-R after drug treatment, again emphasizing that hMφ-R are polarized to an M1-like phenotype.

*In silico* simulations predict that both LUR and TRB induce the upregulation of glycolysis and the PPP. Furthermore, it predicts the production of glycolytic intermediates in the non-oxidative phase of the PPP boosts the glycolysis upon their reincorporation. This increase in the PPP pathway, leads to the generation of NADPH and ROS, as observed in our experiments.

Our results also indicate an increase in the export of citrate to the cytoplasm and the ratio succinate/α-KG, two important features of M1 macrophages (59). Once in the cytoplasm, citrate and succinate act as signaling molecules, thus activating proinflammatory transcription factors such as HIF-1α (6), ROS production (15), and epigenetic regulation linked to an M1 phenotype (60). In contrast, cytoplasmic α-KG is anti-inflammatory, being able to block NF-κB activation (7).

The next step involved the study of glycolytic flux deviation via PHGDH. This enzyme catalyzes the first step of *de novo* synthesis of serine (61), in which the glycolytic metabolite 3-phosphoglycerate is converted into phosphopyruvate, then into phosphoserine and, eventually, into serine. The model predicts that drug treatment of hMφ-R triggers an increased flux throughout the serine biosynthetic pathway. M1 macrophages import newly synthesized serine into the mitochondria, which is then converted into glycine (62) and it is subsequently used for generating glutathione, thus providing hMφ-R protection against ROS. It is also important to highlight that overexpression of PHGDH has been recently related to M2 polarization, as its transcription is triggered following IL-4 stimulation, and its depletion leads to a decrease in the expression of several profibrotic biomarkers (61). However, the PHGDH linkage to a polarization signature might be activity-dependent, as the conversion of phosphopyruvate into phosphoserine requires a nitrogen transference from glutamate, being anti-inflammatory α-KG produced as a result. However, the model predicts that hMφ-R internalize α-KG into the mitochondria to diminish its anti-inflammatory effects (63). This fact is supported by our results.

Lastly, we analyzed *in silico* the role of methylglyoxal synthesis in hMφ-R treated with TRB and LUR. Methylglyoxal is a by-product of glycolysis (64) and is generated from hydroxyacetone (acetol) by acetol monooxygenase (ACTLMO) as the end-product of the catabolism of acetoacetate. Since this compound is highly reactive and generates advanced glycation end-products (AGEs), being metabolized principally by the glyoxalase pathway (two sequential reactions: glyoxalase-1 (GLO1) and glyoxalase-2 (GLO2)) that catalyzes the transformation of methylglyoxal into D-lactate. According to the model, methylglyoxal formation through ACTLMO and degradation through the glyoxalases is increased following both treatments. Interestingly, acetoacetate has been recently reported to confer protection against mitochondrial dysfunction as a consequence of lactic acidosis (65) generated by both methylglyoxal-derived D-lactate and glucose-derived L-lactate (62). Since aerobic glycolysis and methylglyoxal production appear to be upregulated by TRB and LUR; an increase in acetoacetate synthesis, together with lactate conversion into pyruvate *via* lactate dehydrogenase might mitigate the disruption of the mitochondrial function in hMφ-R. In addition, we propose that once in the cytoplasm, citrate might be decarboxylated into acetate, which would likely be used, at least in part, for this purpose.

From a therapeutic point of view, different groups attributed the antitumor effects of TRB and LUR to the fact that these molecules exert a “tropism” for mononuclear phagocytes (monocytes and macrophages) (17), and specifically in tumor-associated macrophages (TAMs) (15). Here, we propose that TRB and LUR promote a proinflammatory M1-like polarization in hMφ-R that retain viability, which could explain additional antitumor activities of these compounds. In this regard, it would be relevant to determine whether there is any correlation between hMφ-R stratification and patients’ outcome to design tailor-made medicine precision early strategies. The next step would be evaluating patients’ immune cells before and after TRB or LUR treatment. Due to these results, it would be promising to evaluate the human lymphoid population's response to these agents to investigate the adaptive immune response, preliminary experiments suggest these molecules modulate lymphoid response and activation state.

## 5 Concluding remarks

TRB and LUR trigger proinflammatory pathways (NF-κB, MAPK, JNK) in hMφ-R and redox biology is altered: NADPH-dependent cytoplasmic and mitochondrial ROS production occurs, and glutathione-related enzymes are overexpressed at 6h which does not depend on the hexose source.

RNAseq shows that TRB and LUR regulate similar hMφ-R biological functions; both molecules upregulate MHC class I protein complexes and lipid catabolic processes which are reflected in the hydrolysis of hMφ-R lipid droplets; the transcription of biosynthetic fatty acid and cholesterol-related enzymes is abolished and specific receptor that internalizes lipids are transcriptionally activated. Lysosomal trafficking is higher when hMφ-R are challenged with either TRB or LUR.

TRB and LUR directly impact mitochondrial hMφ-R physiology; OXPHOS is repressed, and biogenesis is activated as a compensatory mechanism. These facts could be driving the immunometabolic response of this innate immune subset. Finally, TRB and LUR favor glycolysis, PPP and serine biosynthesis *via* the PHGDH pathway, modify TCA cycle and produce methylglyoxal.

## Data availability statement

The datasets presented in this study can be found in online repositories. The names of the repository/repositories and accession number(s) can be found below: NCBI via accession ID 23836796.

## Ethics statement

The studies involving human participants were reviewed and approved by Centro de Transfusiones de la Comunidad de Madrid, under the agreement 28504/000011. The patients/participants provided their written informed consent to participate in this study.

## Author contributions

AP-R. and RL-V designed the study and performed experiments, analyzed data, prepared the figures and revised the manuscript. MM, MF, CA-L, MF-M, JR, SM and AC designed and performed experiments, analyzed data and provided intellectual input and improvements. SS-G, CF, FM and SM provided intellectual input and improvements, discussed results and revised the manuscript. MF and CF developed and applied the GSMM computational analysis. All authors provided intellectual input and revised the manuscript. AP-R, MF, SM, MC and LB discussed the results, wrote the paper, provided funding and intellectual input, and organized the information. All authors contributed to the article and approved the submitted version.
