# Supplementary material for: Immunometabolic actions of trabectedin and lurbinectedin on human macrophages: relevance for their anti-tumor activity
Source: Front Immunol. 2023 Aug 22;14:1211068. doi: 10.3389/fimmu.2023.1211068 (PMC10479946; doi:10.3389/fimmu.2023.1211068)
Supplement: Supplementary file 1 [file DataSheet_1.pdf]

Supplementary material

Supplementary Table 1. Metabolic flux modulation of succinate and α-KG mitochondrial transport by TRB and LUR. Results are shown as the log2FC value of the treatment *vs*. the control for each reaction. Bold values indicate the log2FC of the summatory of each group of reaction fluxes. Identifier: BIGG Models ID.

			**log2FC**	
		**Formula**	**TRB**	**LUR**
**Succinate**	SUCCtm	pi_m + succ_c <=> pi_c + succ_m	-0.4701	-0.6327
	r0819	akg_c + succ_m <=> akg_m + succ_c	0.9629	1.9298
	r0829	so4_m + succ_c <=> so4_c + succ_m	0.4167	-0.9041
	r0830	so3_m + succ_c <=> so3_c + succ_m	0.4612	0.6045
	SUCFUMtm	fum_m + succ_c <=> fum_c + succ_m	0.0543	0.0957
			-1.1134	***-5.9328***
**α-Ketoglutarate**	AKGMALtm	akg_m + mal__L_c <=> akg_c + mal__L_m	0.9935	0.9255
	2OXOADPTm	2oxoadp_c + akg_m <=> 2oxoadp_m + akg_c	0.8480	0.6435
	r2419	akg_c + pi_m --> akg_m + pi_c	1.1553	2.0584
	r2520	akg_m + gthrd_c <=> akg_c + gthrd_m	0.9635	0.9385
	r0819	akg_c + succ_m <=> akg_m + succ_c	0.9629	1.9298
			1.2541	***2.0675***

Supplementary Table 2. mRNA primer sequences (human sequences; 5′–3′).

mRNA	Forward primers sequences	Reverse primers sequences
ARG1	GTCTGTGGGAAAAGCAAGCG	CACCAGGGTGATTCTTCCGT
CD274	TGGCATTTGCTGAACGCATTT	TGCAGCCAGGTCTAATTGTTTT
CXCL10	GTGGCATTCAAGGAGTACCTC	TGATGCCCTTCGATTCTGGATT
FASN	AAGGACCTGTCTAGGTTTGATGC	TGGCTTCATAGGTGACTTCCA
GPR132	TGTTCCAGACGGAAGACAAGG	GCGTAGTAGTACCCGGCAA
HIF1A	GAACGTCGAAAAGAAAAGTCTCG	CCTTATCAAGATGCGAACTCACA
HMGCR	TGATTGACCTTTCCAGAGCAAG	CTAAAATTGCCATTCCACGAGC
HMOX1	AGTCTTCGCCCCTGTCTACT	CTTCACATAGCGCTGCATGG
IL10	CGAGATGCCTTCAGCAGAGT	CGCCTTGATGTCTGGGTCTT
IL10RA	CTGAAGAGCCCCAGTTCCTC	TCCCGCTGTCTGTGCTATTG
IL6	TTCACCAGGCAAGTCTCCTCA	CCTGAACCTTCCAAAGATGGC
KCNA3	AGTTCCGCGACGAGAAGGA	CGAAGAAGGGATCGGAGAAGC
KCNJ2	TTGCTTTGGCTCACTCGCTT	AAACACAGCAGCCCTTACCT
KCNN4	CCAGCCAGCAGTCCAAGTAG	CCCCTTAGCCACATAGGGTG
LDLR	TCTGCAACATGGCTAGAGACT	TCCAAGCATTCGTTGGTCCC
MRC1	CCATCGAGGAAGAGGTTCGG	GGTGGGTTACTCCTTCTGCC
ORAI1	CTTCCTAGCTGAGGTGGTGC	TAAAGATCAGGCCGASGGGC
PDCD1LG2	ATTGCAGCTTCACCAGATAGC	AAAGTTGCATTCCAGGGTCAC
PFKFB3	TTGGCGTCCCCACAAAAGT	AGTTGTAGGAGCTGTACTGCTT
PTGS2	CGCAGTACAGAAAGTATCACAGGC	GCGTTTGCGGTACTCATTAAAA
RPLP0	CAGGCGTCCTCGTGGAAGTGAC	CCAGGTCGCCCTGTCTTCCCT
SCARB1	AATAAGCCCATGACCCTGAAGC	GCCCCACATGATCTCACCC
STIM1	GGATCTCAGAGGGATTTGACCC	TGTCAGGCAGTTTCTCCACC
SUCNR1	GGAGACGTGCTCTGCATAAG	AGGTGTTCTCGGAAAGGATACTT
TLR4	TTTGGACAGTTTCCCACATTGA	AAGCATTCCCACCTTTGTTGG
TNF	CCAGAGGGAAGAGTTCCCCAGGG	AGGCTTGTCACTCGGGGTTCGAG

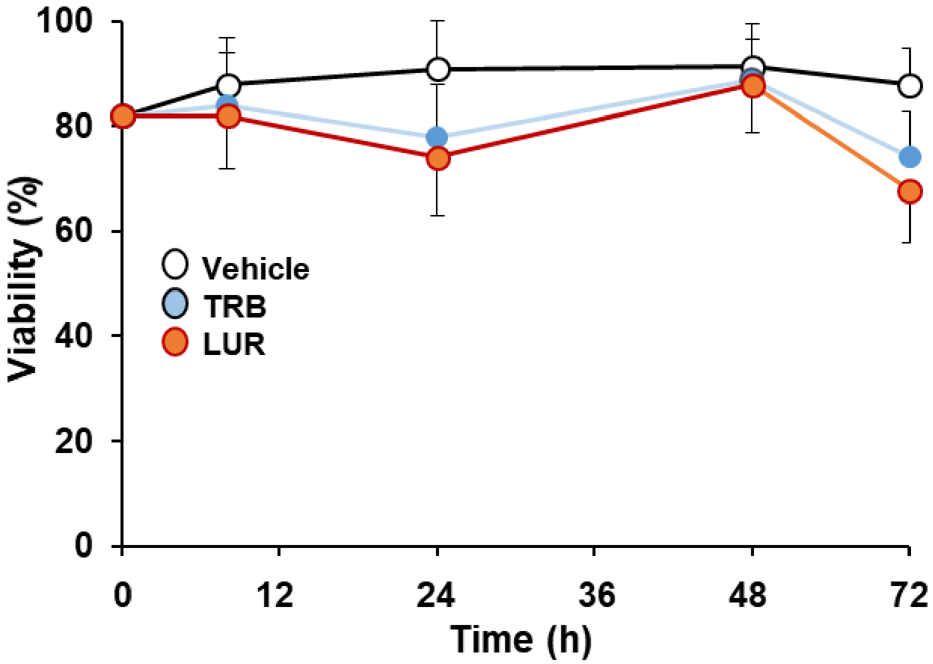

Suppl. Figure S1. Time-course of hMφ-R viability after treatment with TRB or LUR. Cells were treated for the indicated times with 50 nM of the drugs and the cell viability was determined as indicated in Methods. Results show the mean ± SD of 10 different donors. No statistically significant differences were observed.

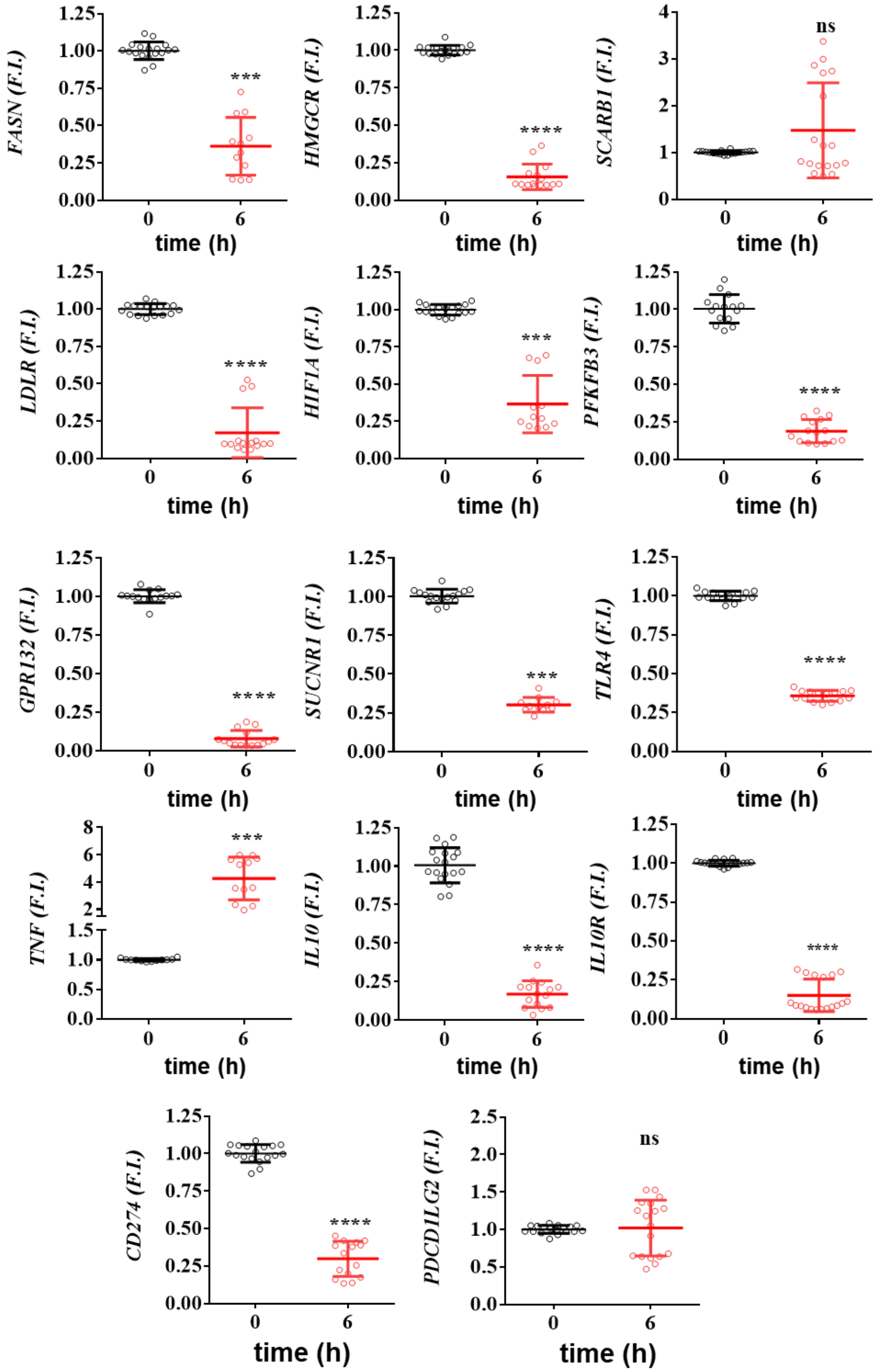

Suppl. Figure S2. hMφ-R differential gene expression time-course patterns after 6 hours of treatment with 100 nM LUR. hMφ-RNAs from healthy donors were isolated and underwent RT-qPCR analysis of the indicated genes. Results show the mean ± SD of fold induction (F.I.) from 6 different donors assayed per triplicate. *p<0.05; **p<0.01; ***p<0.001; ****p<0.0001 *vs*. 0 hours with vehicle. **ns**, not statistically significant.

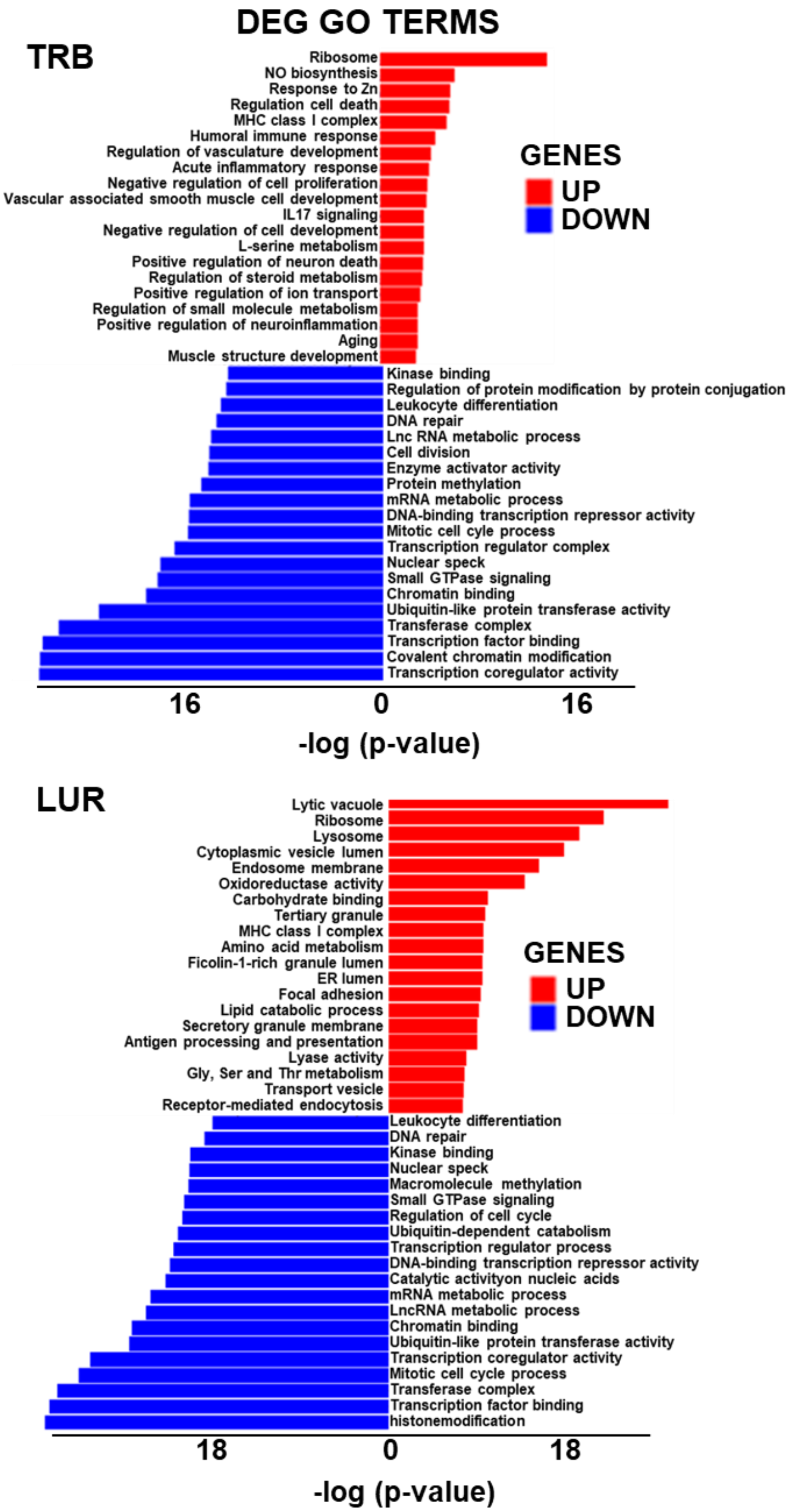

Suppl. Figure S3. RNAseq overlapping transcriptome of hMφ-R after treatment for 6 hours with 100 nM TRB and LUR. Data were obtained after differential gene expression analysis by gene ontology functional annotation**.** Results show the mean log2 of fold induction (F.I.) from 5 different donors *vs*. 6 hours with vehicle.

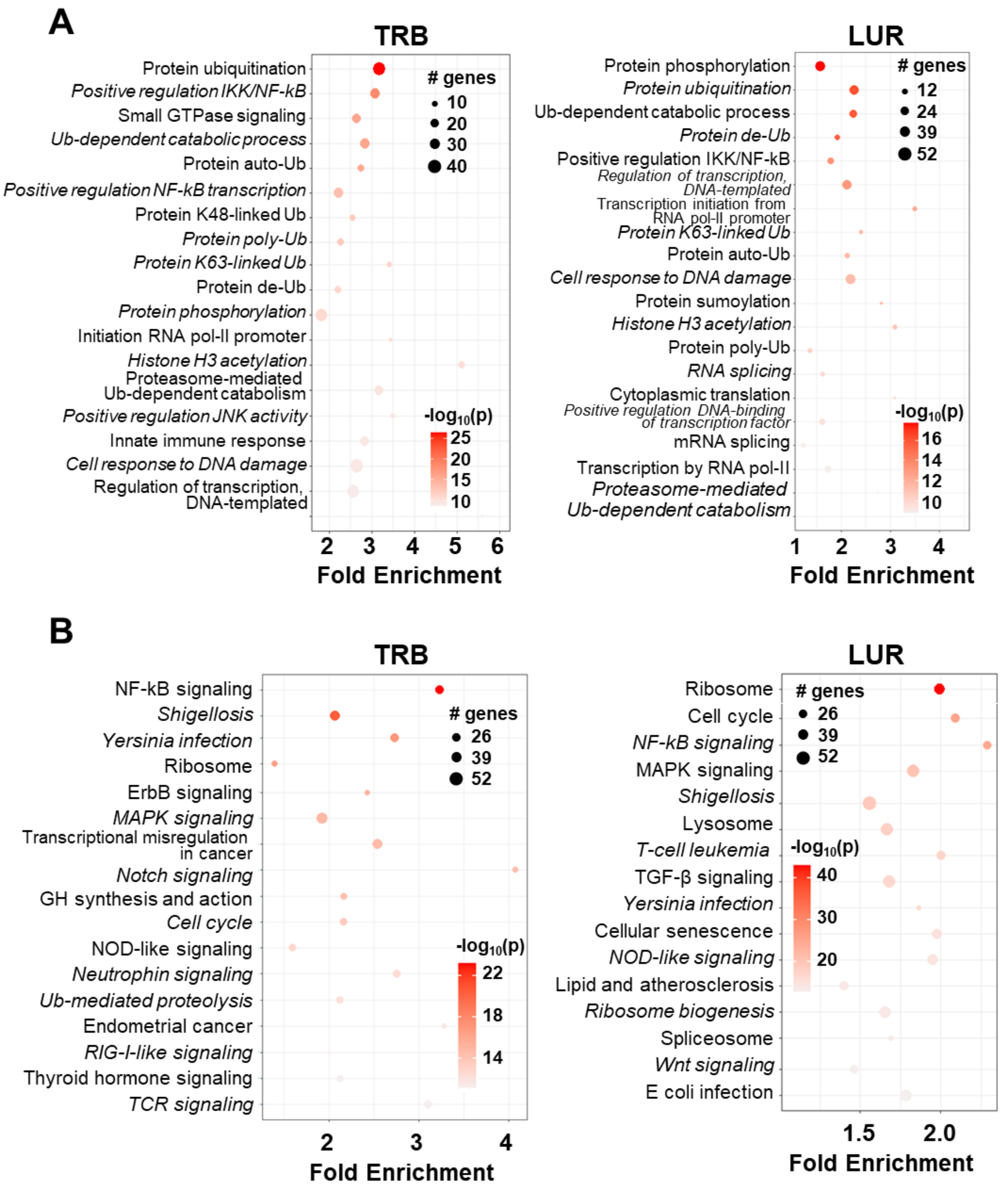

Suppl. Figure S4. Gene ontology functional annotation of biological process in hMφ-R after 6 hours of treatment with 100 nM TRB or LUR (pathfindR). (A) Results show the mean log2 fold of enrichment from 5 different donors *vs*. 6 hours with vehicle. (B). KEGG gene ontology functional annotation of biological process in hMφ-R after treatment with TRB or LUR (pathfindR). Results show the mean log2 fold of enrichment from 5 different donors *vs*. 6 hours with vehicle.

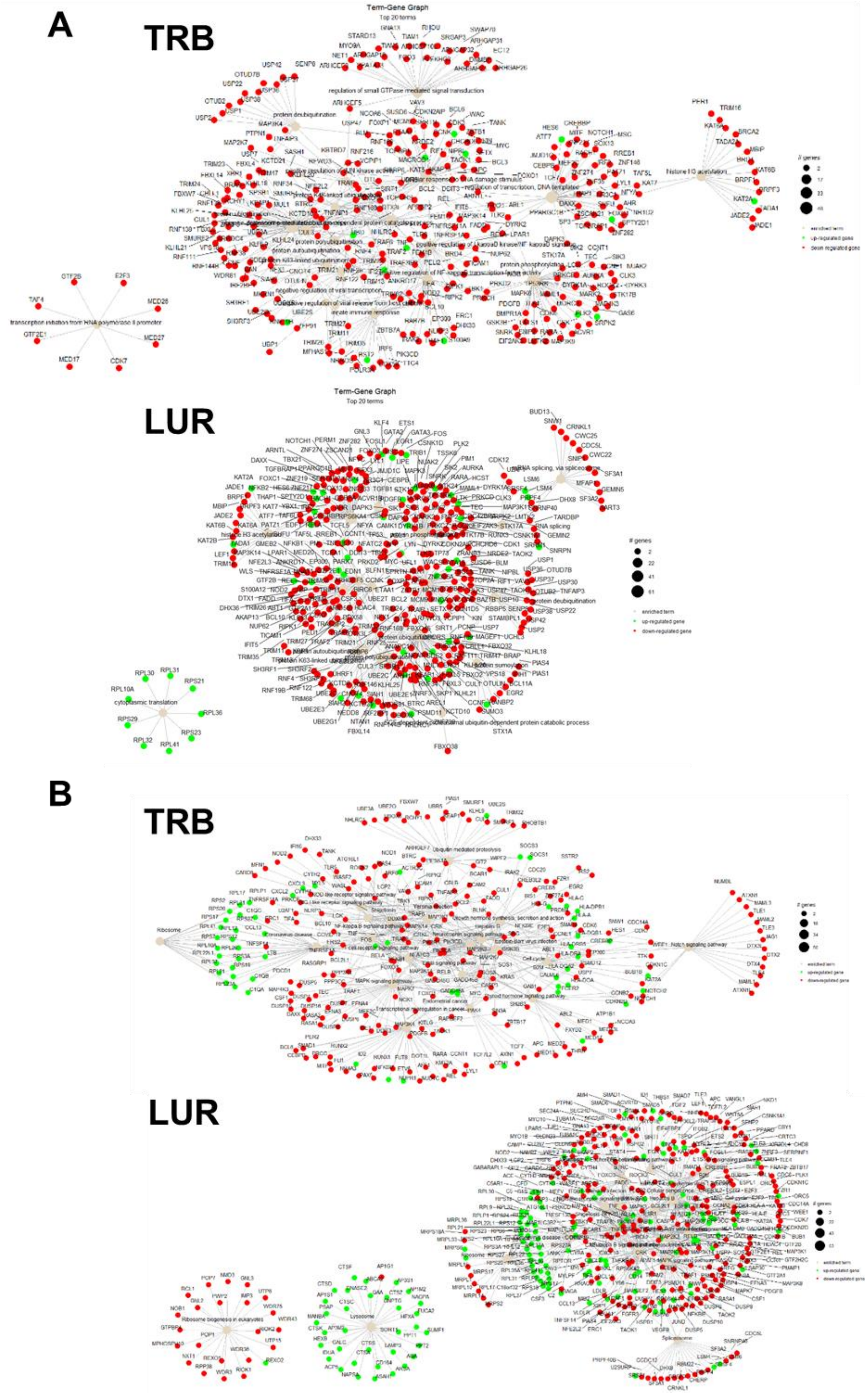

Suppl. Figure S5. Gene ontology functional annotation of biological process in hMφ-R after 6 hours of treatment with 100 nM TRB or LUR (pathfindR). RNAseq analysis from healthy donors. Results show the mean log2 fold of enrichment from 5 different donors *vs*. 6 hours with vehicle.

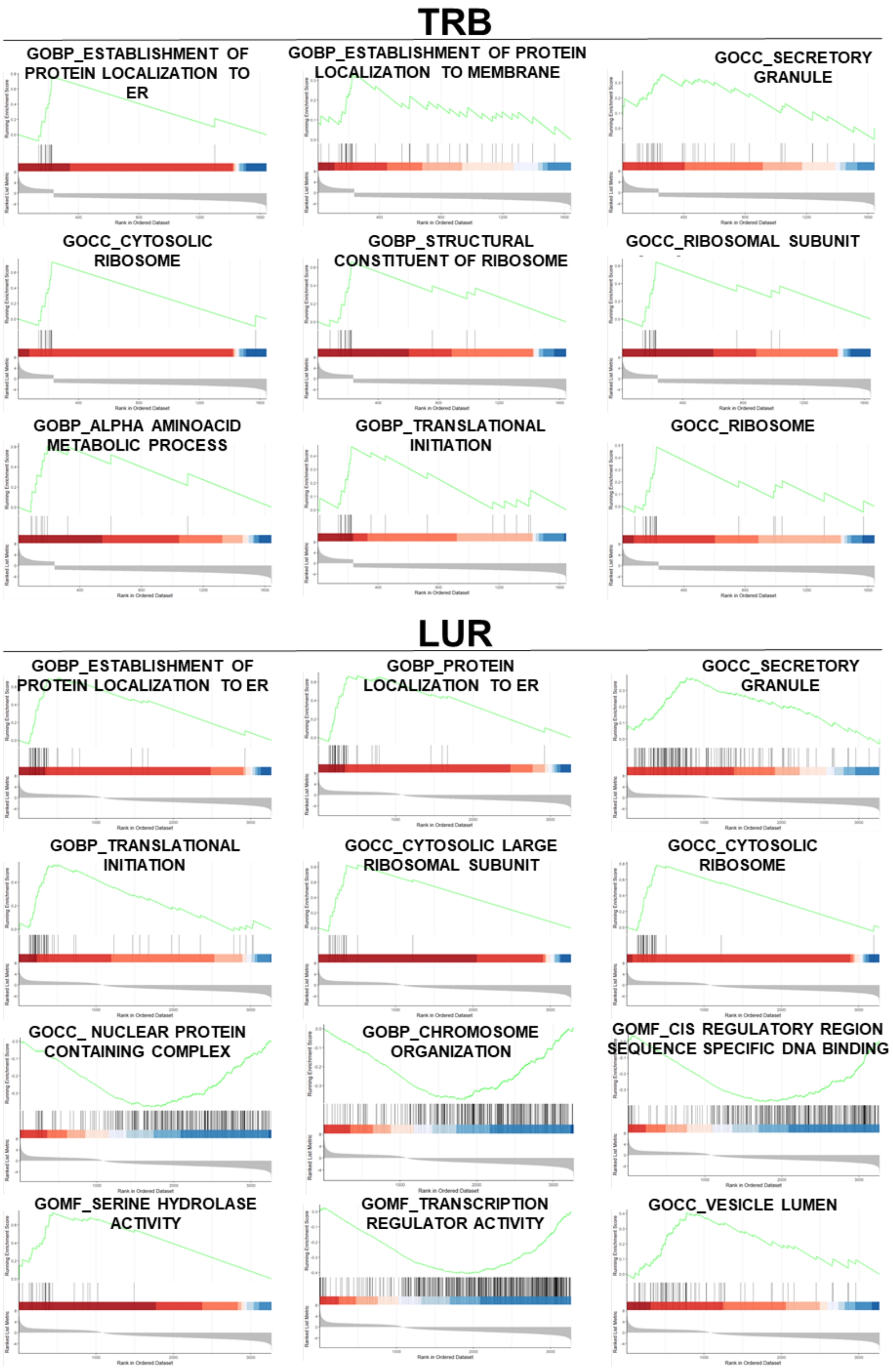

Suppl. Figure S6. RNAseq functional annotation in hMφ-R after 6 hours of treatment with 100 nM TRB or LUR. hMφ-R-RNAs from healthy donors. Results show the mean log2 of fold induction (F.I.) from 5 different treated donors *vs*. 6 hours with vehicle.

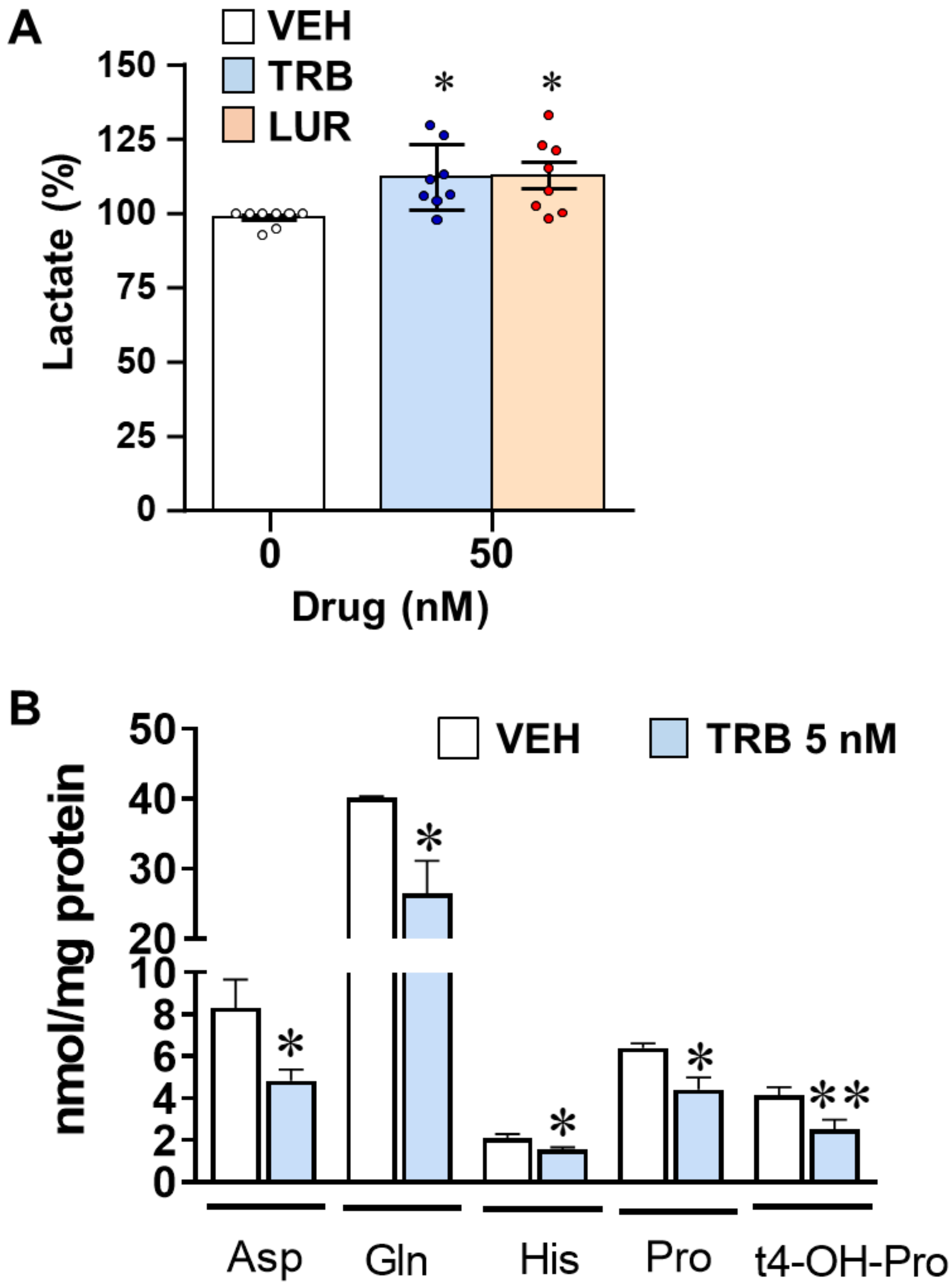

Suppl. Figure 7. Immunometabolic reprogramming of hMφ-R in the presence of TRB or LUR. (A) hMφ-R lactate accumulation in the culture medium after 16h of treatment with 50 nM TRB or LUR. Results show the mean ± SD (% *vs*. untreated cells) from 8 different healthy donors assayed per triplicate. (B) hMφ-R cell pellets were analyzed after 16h of treatment with TRB by BIOCRATES Absolute*IDQ*® p180 kit. Results show the mean ± SD of the aminoacid concentration from 3 different healthy donors. *p<0.05; **p<0.01 *vs*. the vehicle condition.

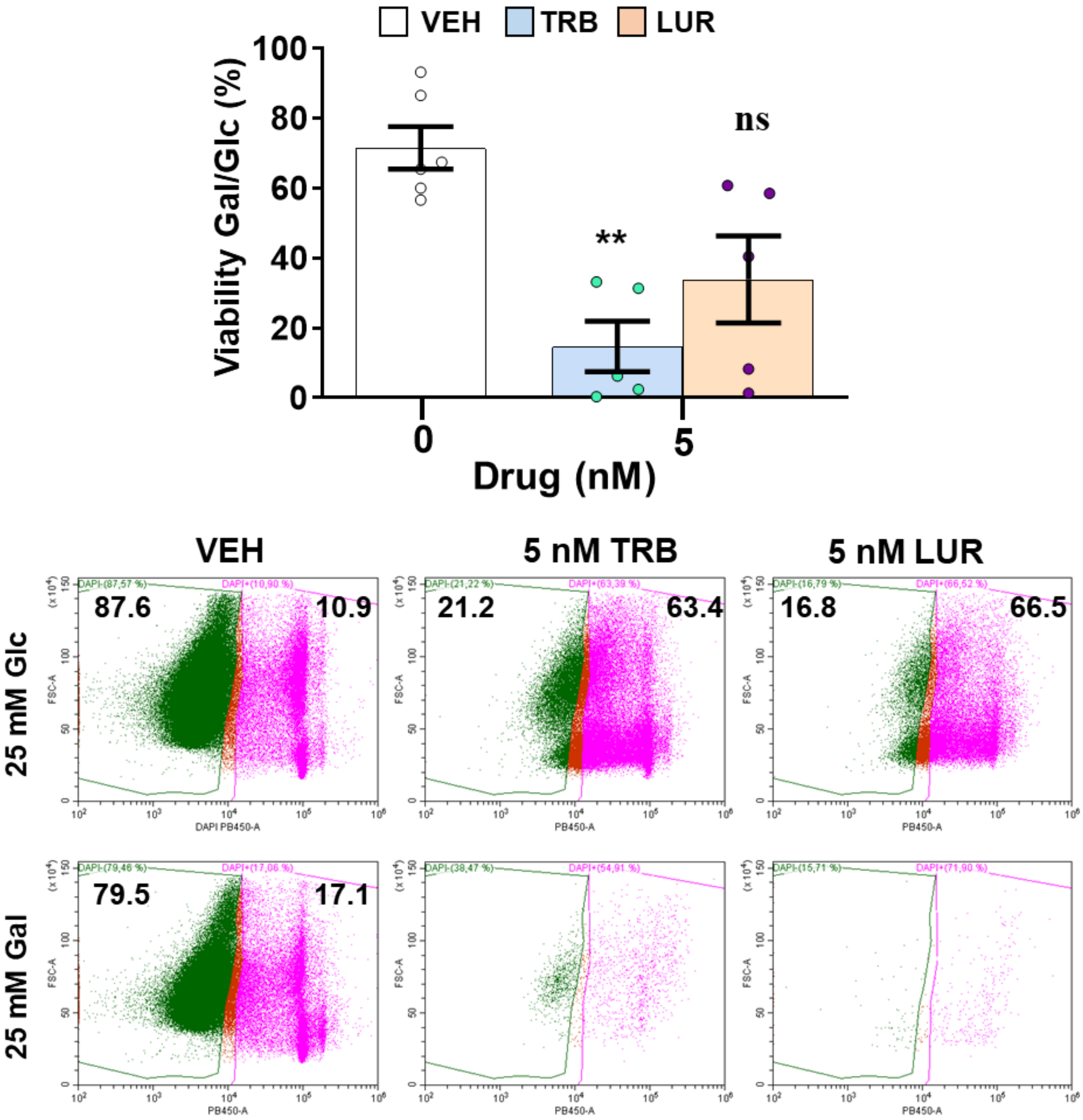

Suppl. Figure S8. Mitochondrial function impairment in hMφ-R maintained in 25 mM glucose (Glc) or galactose (Gal) as hexose source and in the presence of TRB or LUR. hMφ-R were incubated in RPMI1640 medium and Glc or Gal as hexose substrate. Cells were treated for 72h with 5 nM TRB or LUR. Results show the mean ± SD of the indicated parameters (expressed in %) from 6 different healthy donors. **p<0.01 *vs*. the vehicle condition.

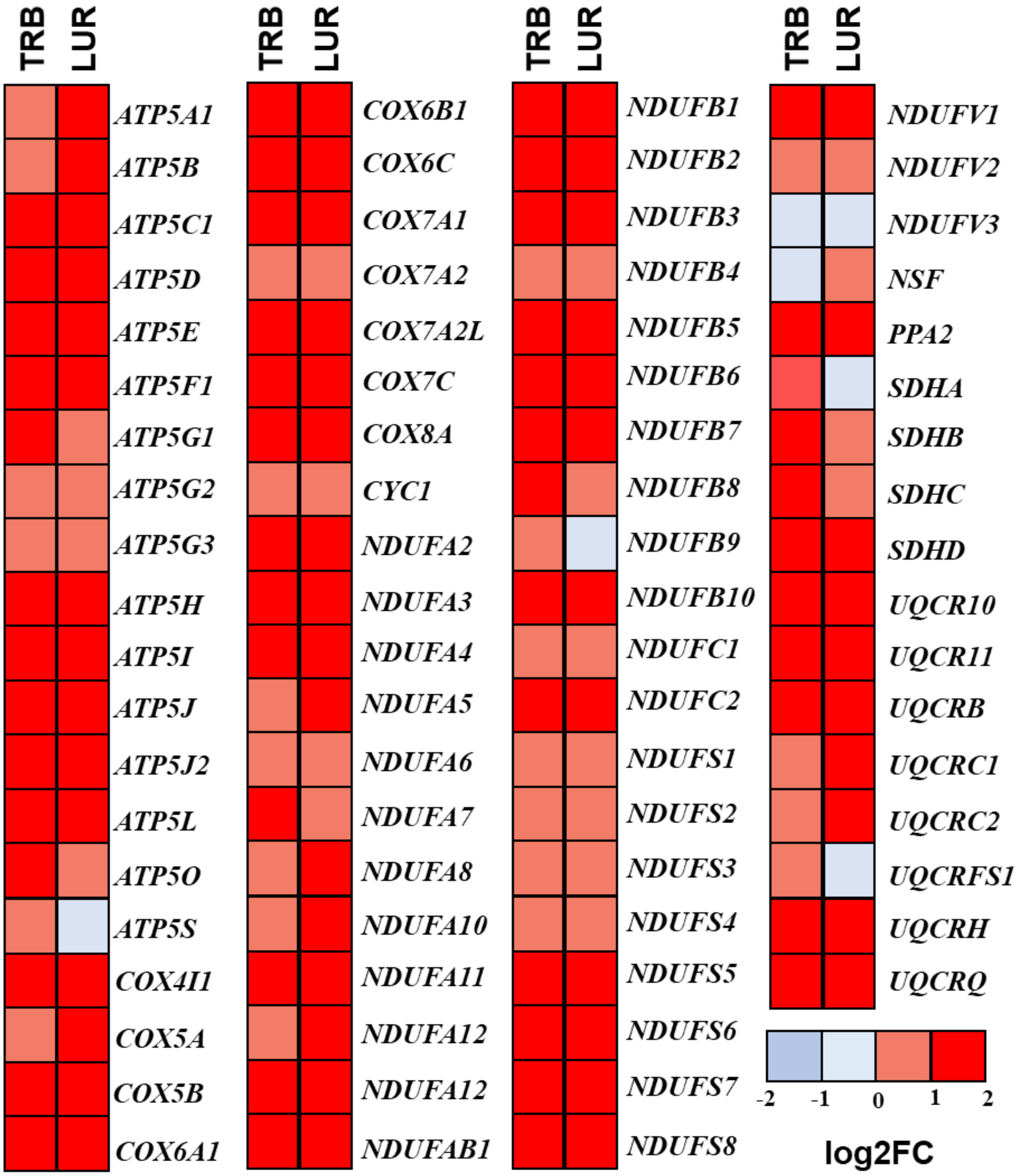

Suppl. Figure S9. RNAseq hMφ-R genes involved in mitochondrial function after treatment of the cells with TRB or LUR. Results show the graphical representation of the abundance of the indicated genes as the mean log2 of fold induction (F.I.) from 5 different donors vs. 6 hours with vehicle

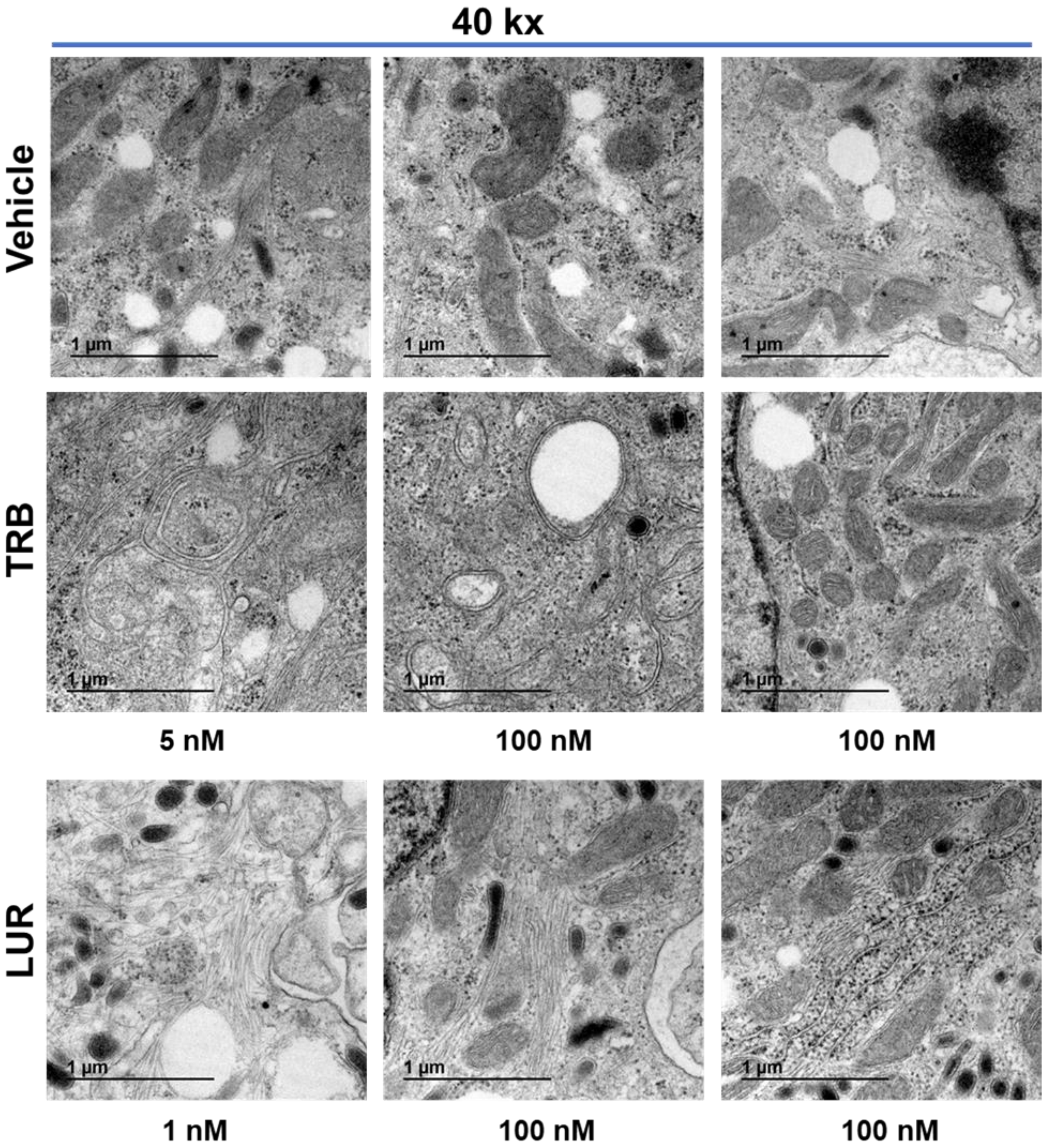

Suppl. Figure S10. Transmission Electron Microscopy of hMφ-R incubated with TRB or LUR. Representative images from hMφ-R cells were analyzed after 24h of treatment with the indicated concentrations of TRB or LUR.

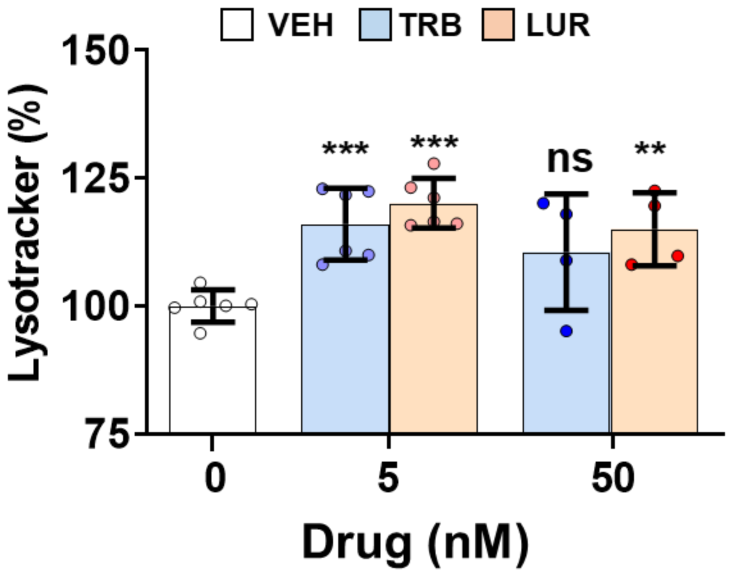

Suppl. Figure S11. Effect of TRB and LUR on the labeling of lysosome trafficking. hMφ-R were treated for 24h with the indicated concentrations of TRB and LUR and the fluorescence of the lysotracker was quantified by flow cytometry as indicated in the Methods sections. Results show the mean ± SD of the indicated parameters (expressed in %) from 6 different healthy donors. $^{**}p<0.01$; $^{***}p<0.001$ *vs*. the vehicle condition.

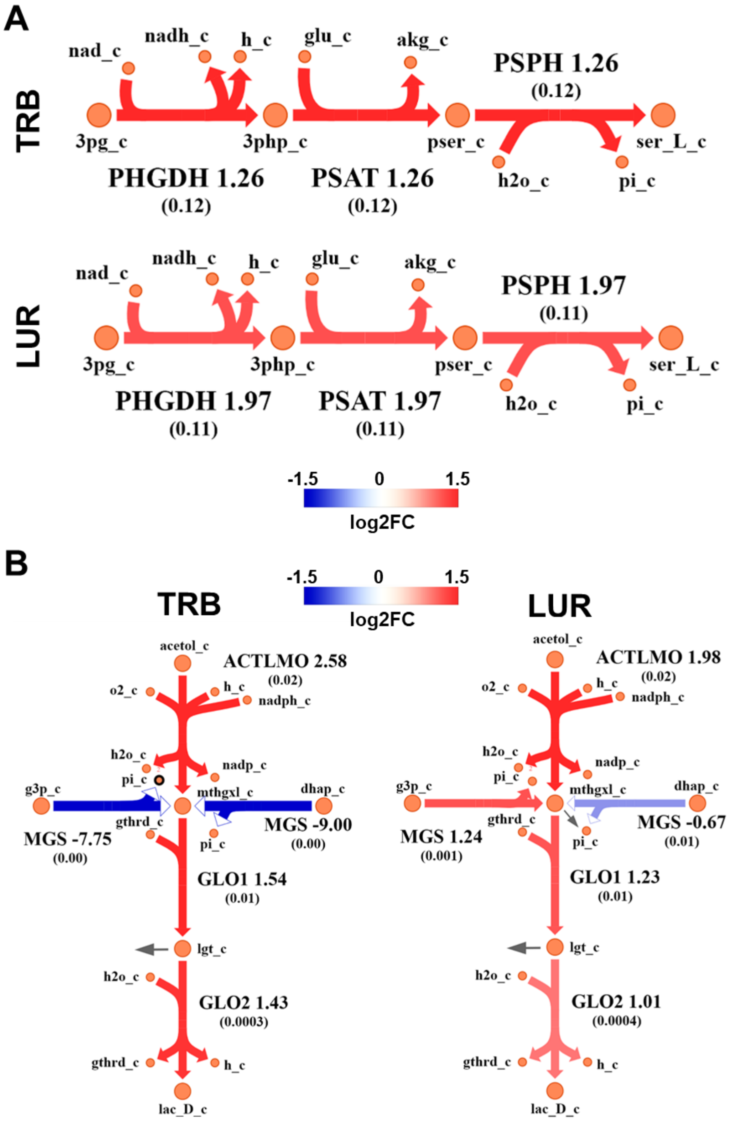

Suppl. Figure 12. Metabolic flux map of TRB and LUR modulation of the PHGDH and methylglyoxal pathways in hMφ-R. (A) Flux upregulation and downregulation are shown in red and blue, respectively. Each reaction is presented as the enzyme/transporter ID, associated log2FC value of the flux, and the predicted flux value (parenthesis). PHGDH: 3-phosphoglycerate dehydrogenase; PSAT: phosphoserine aminotransferase; PSPH: phosphoserine phosphatase; 3pg: 3-phosphoglycerate; 3php: 3-phosphopyruvate; pser: phosphoserine: ser_L: L-serine; glu: glutamate; akg: α-ketoglutarate; pi: inorganic phosphate; nad(h): nicotinamide adenine dinucleotide; _c: cytosolic. (B) Methylglyoxal flux upregulation and downregulation are shown in red and blue, respectively. Each reaction is presented as the enzyme/transporter ID, associated log2FC value of the flux, and the predicted flux value (parenthesis). ACTLMO: acetol monooxygenase; GLO1: glyoxalase 1; GLO2: glyoxalase 2; MGS: methylglyoxal synthesis; mthgxl: methylglyoxal; lgt: lactoylglutathione; lac_D: D-lactate; dhap: dihydroxyacetone phosphate; g3p: glyceraldehyde-3-phosphate; h: proton; nadp(h): nicotinamide adenine dinucleotide phosphate; gthrd: reduced glutathione; pi: inorganic phosphate; _c: cytosolic; _m: mitochondrial. *: In this case, although the log2FC indicates a smaller flux, the direction of the reaction has changed, if compared to the control.
